# Bedroom Concentrations and Emissions of Volatile Organic Compounds during Sleep

**DOI:** 10.1021/acs.est.3c10841

**Published:** 2024-04-24

**Authors:** Betty Molinier, Caleb Arata, Erin F. Katz, David M. Lunderberg, Jennifer Ofodile, Brett C. Singer, William W Nazaroff, Allen H. Goldstein

**Affiliations:** † Department of Civil and Environmental Engineering, University of California, Berkeley, California 94720, United States; ‡ Department of Chemistry, University of California, Berkeley, California 94720, United States; § Department of Environmental Science, Policy and Management, University of California, Berkeley, California 94720, United States; ∥ Indoor Environment Group and Residential Building Systems Group, Lawrence Berkeley National Laboratory, Berkeley, California 94720, United States

**Keywords:** indoor air, VOC composition, residential microenvironments, CO_2_

## Abstract

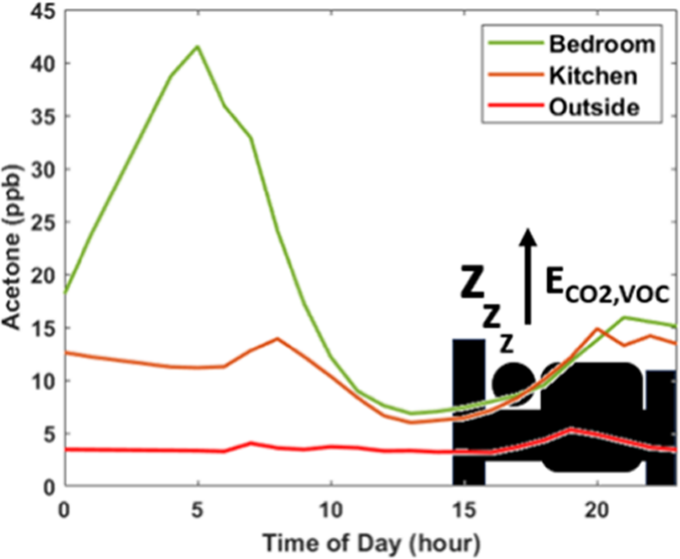

Because humans spend about one-third of their time asleep in their bedrooms and are themselves emission sources of volatile organic compounds (VOCs), it is important to specifically characterize the composition of the bedroom air that they experience during sleep. This work uses real-time indoor and outdoor measurements of volatile organic compounds (VOCs) to examine concentration enhancements in bedroom air during sleep and to calculate VOC emission rates associated with sleeping occupants. Gaseous VOCs were measured with proton-transfer reaction time-of-flight mass spectrometry during a multiweek residential monitoring campaign under normal occupancy conditions. Results indicate high emissions of nearly 100 VOCs and other species in the bedroom during sleeping periods as compared to the levels in other rooms of the same residence. Air change rates for the bedroom and, correspondingly, emission rates of sleeping-associated VOCs were determined for two bounding conditions: (1) air exchange between the bedroom and outdoors only and (2) air exchange between the bedroom and other indoor spaces only (as represented by measurements in the kitchen). VOCs from skin oil oxidation and personal care products were present, revealing that many emission pathways can be important occupant-associated emission factors affecting bedroom air composition in addition to direct emissions from building materials and furnishings.

## Abstract 2

Examination of VOC composition in residential bedrooms is limited. This study reports peak average concentrations and emission rates of a wide range of VOCs in the bedroom relative to the rest of the house.

## Introduction

On average, Americans spend their time indoors and 70% in residences.^1^ Bedrooms, where humans spend about a third of their time, are often not well-ventilated, leading to the accumulation of indoor emissions and increased exposure. Some studies have investigated the impact of exposure to dust, carbon dioxide (CO_2_), total volatile organic compounds (VOCs), and other airborne species in the bedroom,^2−4^ as well as effects of indoor air composition on sleep quality and subsequent cognitive ability,^5−9^ but VOC composition in normally occupied bedrooms during sleep has yet to be characterized.^4^ Improved understanding of VOC concentrations during sleep, including those resulting from human emissions, has implications for determining cumulative inhalation exposures and potential effects on public health as well as indoor-to-outdoor transport and impacts on atmospheric chemistry.

VOCs represent an important aspect of indoor air quality. This chemical class includes species known to be irritants and carcinogens.^10^ Ventilation is an effective mechanism for removing pollutants emitted from indoor sources and thereby limiting exposures, but it also increases outdoor-to-indoor pollutant transport, making it undesirable in polluted areas. VOCs also play a major role in atmospheric chemistry as their oxidation can lead to the formation of ozone, secondary VOCs, and secondary organic aerosol (SOA),^11^ all of which have implications for air pollution and public health. As such, it is important to identify sources of residential indoor air pollutants in order to understand and, when necessary, mitigate human exposures and associated adverse health risks.

Concentrations of many VOCs are higher indoors than outdoors. Indoor emissions have been studied in many settings, including grade school and university classrooms,^12−16^ museums,^17^ office and commercial buildings,^18,19^ stadiums,^20^ and residences.^21−23^ Known VOC sources include building materials, cleaning and personal care products, furnishings, human activities (e.g., cooking), and outdoor air.^22^ A significant indoor source is human occupants themselves, as VOCs have been detected in breath and skin.^24^ More than 1800 VOCs have been detected in at least one type of human effluent; these species are commonly classified as “bioeffluents,” the most abundant of which are acetone and isoprene.^24,25^ Carbon dioxide (CO_2_) is also an important bioeffluent in breath and a useful indicator of the presence of human emissions indoors, including VOCs. Bioeffluent and other VOC emissions can vary with age, exercise, stress, clothing, presence of ozone or other oxidants, and building characteristics (temperature, relative humidity, etc.).^26−31^

Inhalation exposure to VOCs in indoor spaces can have health consequences. Some studies have suggested that exposure to VOCs that have been characterized as bioeffluents in combination with 3000 ppm of CO_2_ can negatively affect cognitive abilities.^32,33^ Individuals report experiencing irritation at exposure to high levels of VOCs and bioeffluents,^34^ although individual thresholds for irritants are variable.^35,36^ Additionally, some studies note little to no change in cognitive ability as a result of exposure to breath emissions,^34,36^ or attribute those changes to other factors,^33^ indicating a gap in quantitative understanding of the impacts of VOC exposure on human health.

Many studies have reported direct breath, skin, and whole-body VOC composition and emission rates in clinical and laboratory settings^36−39^ and have explored in situ VOC sources and abundance in residences.^21−23^ To the best of our knowledge, there are no prior studies characterizing VOC composition in a bedroom during sleep in a normally occupied residence. We explore the accumulation of VOCs overnight and determine corresponding bedroom air change rates and VOC emission rates. All of these components are key to understanding the role of human activity and ventilation in VOC emissions and their impact on indoor air quality.

## Materials and Methods

### Site Information

Measurements were conducted at a normally occupied (defined here as a dwelling in which the current occupants have been residing for enough time to establish regular patterns of behavior that influence emissions) single-family residence (designated “H3”) in Oakland, California. Sampling was undertaken between October 01 and December 06, 2021. This paper focuses on the last 3 weeks of measurements, which included measurements made in an occupied bedroom during overnight periods. The two-story residence was originally built in 1910, expanded in 1960, and retrofitted for energy efficiency (envelope air sealing and duct sealing) in the 2010s. The house has a kitchen, living room, dining room, three bedrooms, office, lower-level recreational room, garage, and attic. Excluding bathrooms, the flooring in the living space where measurements were made is finished hardwood. A schematic floor plan of the residence can be found in the Supporting Information (Figure S1). The living space volume was estimated to be ∼390 m^3^. Regular occupants (defined here as individuals residing in the home full-time) were two adults and a cat with occasional overnight guests. Indoor activities (defined here as any activity conducted within the home as specified by the occupants) include cooking, exercising, hosting social gatherings, and professional cleaning. Occupants maintained daily recorded logs of their presence and activities at the house. The H3 site was heated by a forced air natural gas-fired furnace, and occupants were cooked with a natural gas oven and stove (equipped with continuous pilot lights), sometimes employing the range hood for ventilation. The storage water heater and clothes dryer in the garage also used natural gas and had pilot lights. For this study, instruments for measuring VOCs, CO_2_, and ozone were located in the basement/garage, and metadata sensors (motion, temperature, humidity, additional CO_2_ sensors) were deployed throughout the house. Tracer gases were continuously released into the attic (700 ppm of propene-*d*_6_, released at 11.3 mL min^–1^), living zone (1000 ppm of butene-*d*_3_, released at 11.3 mL min^–1^), and garage (1075 ppm of propene-*d*_3_, released at 14.3 mL min^–1^) to determine the air change rate of the house and also to characterize interzonal flows.

### Measurements

Air was sampled continuously, cycling among five indoor locations and one outdoor location at ∼2 l/min through poly(tetrafluoroethylene) (PTFE) sample lines (6.35 mm od × 3.175 mm id), each of ∼23 m length with a 2.0 μm pore size 47 mm PTFE filter at the inlet. The six lines were subsampled sequentially for 5 min each using automated switching valves, resulting in two complete measurement cycles per hour. This work primarily focuses on measurements from the bedroom, kitchen, and outdoor sampling locations. The bedroom sampling line was originally located in the living room but was moved several weeks into the measurement campaign. A proton-transfer reaction time-of-flight mass spectrometer (PTR-TOF-MS, Ionicon Analytik GmbH, Austria, PTRTOF 8000) was used to measure VOCs through soft chemical ionization via hydronium ions (H_3_O^+^) to facilitate their protonation.^13^ The instrument was calibrated nightly during the hours 01:00–04:00, alternating between two standard calibration gases from Apel-Riemer of known composition, pressure, and flow rate. Standard gas compositions can be found in Tables S1 and S2. The net positive charge of protonated ions enabled detection in the TOF chamber. Each pulse through the TOF produced a complete mass spectrum. Drift pressure was maintained at 2.2 mbar and E/N at ∼125 Td. Processing this information with the PTRwid package in IDL enabled the detection of mass peaks and the calculation of signal strength in counts per second (cps) for each molecule. Tools such as PTR libraries^40^ were useful in matching chemical formulas of known peaks to those detected in each data set. Signals were converted from cps to parts per billion (ppb) via calibration-derived sensitivity factors or known PTR reaction rates. Signals with a time-average concentration below ∼5 ppt were not considered in the analysis, and both fragments and isotopes were combined with the signals of their parent ions to reduce the VOC data from 433 individual ions to 239 signals (organic ion formulas). Best-estimate compound name assignments are reported together with the corresponding ion formula and observed ion mass (mass-to-charge ratio, *m*/*z*). The high mass resolution (*m*/Δ*m* = 8000) enables the PTR-TOF-MS instrument to distinguish compounds with nominally similar molecular masses but different chemical compositions. We acknowledge the unavoidable limitation of being unable to differentiate among isomers so that, for example, terpenes are necessarily reported as a chemical group rather than as a series of differentiated species. More details regarding instrumental methods can be found in Liu et al.^21^ and Holzinger.^41^ Instruments measuring ozone (ThermoScientific 49i) and CO_2_ (LICOR LI-820) were connected to the same sampling manifold to provide two measurements per hour from each of the six sampling locations.

### Analysis Methods

Measurements taken by the PTR-TOF-MS and the LICOR at 1 s resolution were averaged over each minute. The last minute of each 5 min sampling period at each location was used in analyses reported here, ensuring minimal interference from valve switching and resulting in one average data point every 30 min per location. The time series of data points at each location were linearly interpolated to enable synchronized comparison, for example, between the kitchen and the bedroom. Individual CO_2_ sensors (Netatmo Weather Station) measured concentrations in specific rooms (kitchen, main bedroom, lower level, living room, garage, second bedroom) recorded at 5 min resolution and averaged over 30 min periods, most of which matched a VOC sampling location.

Figure S2 compares LICOR and Netatmo CO_2_ data in the main bedroom over a three-week period. The LICOR was limited to a maximum concentration of approximately 2000 ppm, whereas the Netatmo sensor was able to measure concentrations above 3000 ppm, such that only Netatmo observations were reliable at concentrations above 2000 ppm. An orthogonal regression analysis was performed in MATLAB between the two sets of data below 1800 ppm, and the resulting corrections were applied to improve the accuracy of the Netatmo data. More details can be found in the Supporting Information (SI). The CO_2_ enhancement during the sleeping periods, or periods during which occupants reported being indoors and asleep in their presence logs, are reported in the SI. The corrected CO_2_ data from the Netatmo sensor in the main bedroom was used in all further computations, which are discussed in the next paragraphs and in the SI.

There were 20 nights over which data in the bedroom were collected, two of which were vacant days. During these vacant periods, the regular occupants were away from the study site. However, because the researchers used these days to conduct manipulation experiments, the data were not considered representative of true vacant conditions. As demonstrated in Figure S3, the pattern of overnight accumulation for the remaining 18 occupied nights was variable in the CO_2_ concentration profile. The likely cause of these differences is that the exterior windows or bedroom doors were open on some but not all nights during the sleeping period. To highlight the lower-ventilation conditions in the analysis to follow, the nights with substantial CO_2_ accumulation were identified and emphasized. This approach also stresses circumstances in which the bedroom air composition is substantially decoupled from that in the remainder of the house. Overnight accumulation of CO_2_ in units of ppm·h was determined for all 18 occupied nights, as summarized in Table S3. Briefly, the background concentration was subtracted and estimated as equal to the fifth percentile of CO_2_ concentrations over all 18 nights, and the area under the nightly background-subtracted curve was calculated using the “trapz” function in MATLAB. The duration of each curve of interest was consistent with the length of time over which the CO_2_ concentration profile was elevated significantly above the concentrations in the kitchen. Six nights with an accumulation of CO_2_ below 1000 ppm·h were excluded, and the remaining 12 nights were used for further analysis: November 18, 23, 25–28, 30, December 01–03, 05, and 06. Of the six nights that were removed from the analysis set, four had only one occupant in the bedroom. The remaining two nights likely had lower CO_2_ accumulation resulting from higher ventilation in the bedroom. By this approach, the conditions in which the overnight bedroom air composition is potentially strongly influenced by occupant bioeffluents are emphasized.

Time-averaged concentrations for each night were calculated over the period during which occupants were physically in the bedroom, as determined by the activity and presence logs. A 3 h peak period, beginning at the time that the peak in signal was reached each night, was selected as the averaging period for each of the 12 nights. All 12 average peak concentrations for each VOC for the bedroom, kitchen, and outdoors were determined and subsequently reported in Table S4. It is important to note that this peak occurred during the calibration period on Nov 23 and 25. Because no concentration data were acquired during calibration, the averaging period on those days includes linearly interpolated data. Additionally, the concentration ratios comparing 3 h peak bedroom and kitchen air (*C*_bed_/*C*_kitch_) as well as comparing bedroom and outdoors (*C*_bed_/*C*_out_) are reported. Only VOC concentrations with an average concentration above 0.005 ppb (the reporting limit for the PTR-TOF-MS) and with *C*_bed_/*C*_kitch_ ≥ 1.30 (see Table S4) are noted. An enhancement threshold of 30% enables analysis of compounds whose behavior is considerably influenced by the bedroom microenvironment at the study site and excludes compounds at concentrations averaging below the detection limit.

The air change rate (ACR) of the bedroom is important for estimating emission rates and must be determined for the room itself rather than the whole house, as its doors and windows are often closed, leading to a decoupling of this room from the rest of the house. Bedroom ACR, *A*, is determined using eq 1,(44) where ER_CO_2__ refers to the emission rate of CO_2_ in grams per hour (g h^–1^), Δ*C* and *C̅* are the change in concentration and average concentration, respectively, over some duration Δ*t* (defined here as the time (*t* = 0) between when the occupants went to sleep and the time (*t* = Δ*t*) of the peak in bedroom CO_2_ concentration), and *V* is the volume of the room, which is estimated to be 37 m^3^. To be dimensionally consistent, CO_2_ concentration terms are converted from ppm to g m^–3^ units using the ideal gas law at standard temperature and pressure conditions (STP).
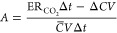
1

Emission rates of VOCs are important for use in emission inventories and modeling. Developing estimates for these factors can be useful to characterize how indoor emission sources affect air composition and chemistry. The bedroom ACR was used to estimate VOC emission rates for all compounds with an average concentration of above 0.005 ppb and with *C*_bed_/*C*_kitch_ ≥ 1.30 (see Table S4). Mass emissions (*E*, μg) are calculated according to eq 2,^21,22^ where Δ*C*_*i*_ and *C̅*_*i*_ are the change in concentration and average concentration of species *i*, respectively, over some sleeping period Δ*t*_sleep_ (defined as the time (*t* = 0) between when the occupants went to bed and the time (*t* = Δ*t*_sleep_) the occupants recorded waking up), *A* is the ACR in h^–1^ as calculated in eq 1, and *V* is the volume of the room in m^3^.
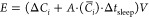
2

To be dimensionally consistent, VOC species *i* concentration terms were converted from units of ppb to μg m^–3^ using the ideal gas law at STP. Total emissions (μg) were determined for each of the 12 analyzed nights and then normalized by duration of sleep per the activity log for two bounding assumptions: (1) bedroom air exchange was with outdoor air only and (2) bedroom air exchange was with other indoor air only (as represented by data from the kitchen) in order to establish a range of plausible estimates for these parameters. A material balance box model with concentrations from the bedroom, kitchen, and outdoor sampling locations as inputs was implemented for this analysis. This procedure resulted in 12 lower and 12 upper emission rate (μg h^–1^) estimates that were then averaged and reported in Tables 2 and S5 for relevant VOCs.

## Results and Discussion

### VOC Concentrations Overnight

**Figure 1:**
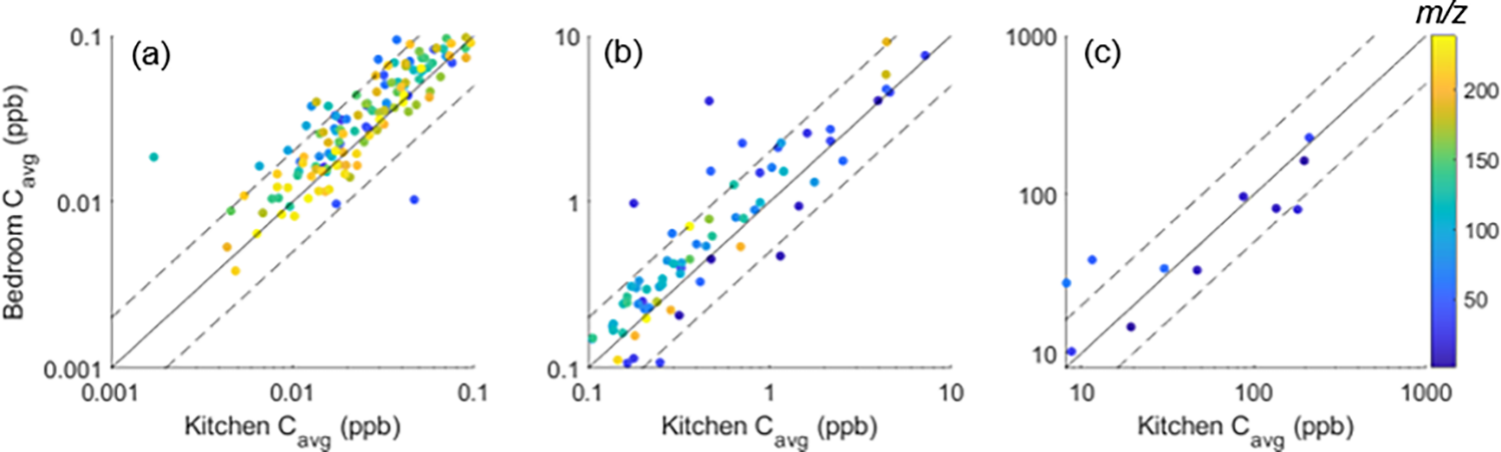
Scatter plots of averaged 3 h peak VOC concentrations over 12 nights in the bedroom and kitchen. Panels (a–c) represent different species concentration ranges. Each point represents one VOC ion. Colors are indicative of mass-to-charge ratios (*m*/*z*), corresponding to the species molecular mass in g/mol, with blue indicating lower *m*/*z* and yellow indicating higher *m*/*z*. The solid line in each panel indicates a 1:1 concentration relationship, with dashed lines representing the 2:1 and 1:2 concentration ratios.

Indoor air composition can change by location in a house depending on occupant behavior and activities, as well as variation in furnishings, surface emissions, and ventilation. By definition, different rooms have different purposes, so the activities conducted in each room vary, leading to a myriad of sources and emissions. While VOCs may be transported from room to room and similar compounds may be emitted from different sources, there is often a clear distinction between emissions from activities such as cooking (kitchen) and the application of personal care products (bedroom or bathroom) during the day. At night, it is expected that occupants will be in their bedrooms, conducting little to no activity aside from sleeping, although this pattern too can vary among individuals. Figure 1 compares the concentrations of VOCs in the occupied bedroom and the unoccupied kitchen, two closely located rooms at H3. The presented concentrations are averaged over an aggregate of 36 h, representing 3 h bedroom peak periods for each of the 12 occupied nights during which time occupants were reported to be present and asleep based on their activity logs. Because the authors relied solely on activity logs provided by the occupants to determine sleeping periods, there are uncertainties around the exact time at which the occupants went to sleep, the actual duration of their sleeping period, and whether or not doors and windows were open. The median relative standard deviation (RSD) for peak bedroom and kitchen concentrations was 42%, and the interquartile range for peak bedroom (kitchen) concentrations was 32–58% (28–69%). Because peak periods were used rather than the entire duration of bedroom occupation, sufficient time had passed to minimize the effects of prior occupation in the kitchen. This approach utilizes an assumption that kitchen concentrations during these peak overnight periods are representative of background unoccupied levels. A similar analysis was conducted for daytime periods (12:00–15:00) over the same dates. That assessment revealed that VOC concentrations were more tightly clustered than during the nighttime hours around the 1:1 line with respect to kitchen average concentrations. In other words, the enhancement of VOC levels in the bedroom was not as significant during the day. The effect of occupant presence on indoor air composition in the bedroom microenvironment is substantial in terms of the number of enhanced compounds and the magnitude of enhancement.

Panel (a) indicates an enhancement of higher molecular weight VOCs in the bedroom at night, albeit at low concentrations (<0.1 ppb). It should be reiterated that the VOC reporting limit is 0.005 ppb. A clear minority of ∼30 compounds are shown to be below the 1:1 line, whereas a large majority (∼96 compounds) are above the 1:1 line, of which 9 are above the 2:1 line. Only one compound was below the 1:2 line. The remaining compounds were very close to the 1:1 line. Panel (b) provides similar information for compounds with intermediate molecular weights and higher concentrations (0.1–10 ppb). For this group, 16 compounds are clearly below the 1:1 line, whereas about 55 compounds are above the 1:1 line, of which eight are on or above the 2:1 line. (Two compounds were below the 1:2 line.) Panel (c) shows only 11 lower molecular weight compounds measured at relatively high concentrations with a nearly balanced distribution with regard to the 1:1 line. Altogether, among the 239 VOC concentrations analyzed at H3, only 21% of them are enhanced in the kitchen at night, presumably due to higher emissions in the kitchen or activities from earlier in the day. Compounds near the 1:1 line are likely related to building emissions or air exchange between rooms. An important finding is that most of the measured VOCs (about 66%) are enhanced in the bedroom overnight due to the accumulation of VOC emissions from various sources, including occupants, when the bedroom is closed. Details related to this finding are explored further in the next sections.

### Quantifying Overnight Enhancement

Average peak concentrations in three sampling locations (bedroom, kitchen, and outdoors) over 12 occupied nights for quantified VOCs can be found in Table S4. The ratio of *C*_bed_/*C*_kitch_ averaged for the three peak hours of 12 occupied nights is also included, with 10th, 25th, 50th, 75th, and 90th percentile values of these ratios being 0.75, 0.96, 1.23, 1.58, and 2.0, respectively. These percentile values indicate that nearly 75% of VOC concentrations were enhanced (*C*_bed_/*C*_kitch_ > 1.0) in the bedroom overnight compared to the kitchen. Indoor-to-outdoor (I/O) ratios for the bedroom are also presented. The ratios in the bedroom with respect to the kitchen concentrations of CO_2_ and O_3_ during the occupied nights were 2.5 and 0.86, respectively. Using a threshold criterion of *C*_bed_/*C*_kitch_ ≥ 1.30 to indicate a substantial enhancement, we find that 94 compounds meet this criterion for being elevated in the bedroom compared with elsewhere in the house during the occupied overnight periods. Some of these elevated compounds may be emitted from occupants themselves; other potential contributors include emissions from surfaces or materials in the bedroom or transport from a room that is strongly decoupled from the kitchen, such as the attached bathroom, as seen in the H3 floor plan diagram (Figure S1).

**Figure 2:**
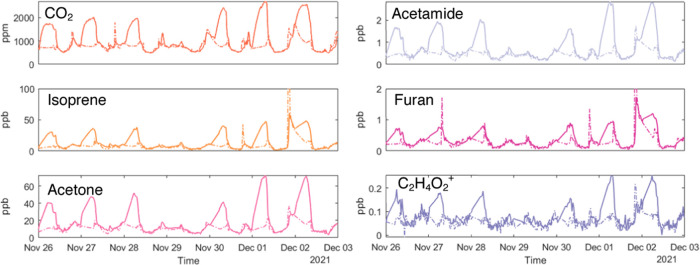
Time series of six compounds in the bedroom (solid) and kitchen (dashed) for a week-long period, showing similar accumulation patterns in the bedroom at night: CO_2_, isoprene, acetone, acetamide, furan, and C_2_H_4_O_2_^+^.

To investigate further, a correlation analysis between CO_2_ and the VOC concentrations detected by the PTR-TOF-MS was conducted over the 12 sleeping periods to determine which VOCs correlated best with a known human breath tracer. The time series for CO_2_ and the five best-correlated VOCs in both the bedroom and kitchen are presented in Figure 2. A total of 25 compounds correlated with CO_2_ at an *R*-value higher than 0.70 (i.e., *R*^2^ greater than about 0.50). No compound had an *R*-value less than or equal to −0.70, indicating no strong anticorrelation with respect to CO_2_. The lack of anticorrelation implies that none of the detected VOC concentrations exhibited opposing behavior to the CO_2_ concentration profile and, therefore, were neither consumed nor lost as CO_2_ was emitted. The average temperature, relative humidity, and absolute humidity in the bedroom over the 12 examined nights were 20 ± 0.63 °C, 57 ± 3.4%, and 10 ± 1 g m^–3^, respectively. CO_2_ did not demonstrate a strong correlation with temperature or absolute humidity, though it was near the threshold with respect to relative humidity (*R* = 0.68).

Time series data for the bedroom and kitchen locations have two measurement results per hour, in accordance with the sampling scheme discussed previously. In terms of correlation with CO_2_, isoprene and acetone ranked first (*R* = 0.97) and second (*R* = 0.95), respectively, followed by acetamide, furan, and *m*/*z* = 60.020 (tentatively identified as the acetate ion, C_2_H_4_O_2_^+^). Isoprene is produced in the human body via the mevalonate pathway, while acetone is produced in a ketone-body formation pathway associated with burning fat (rather than sugar); both are known to be the most abundant bioeffluents emitted in human breath after CO_2_.^24,27,42^ Acetamide and furan are biomarkers for ingested food or beverages, such as coffee or beets.^43^ The remaining compound C_2_H_4_O_2_^+^ presented in Figure 2 was not found in the Human Metabolome Database^43^ but has been reported in other studies involving PTR-TOF-MS.^14^ Other compounds that correlated well with CO_2_ have isomers that could be either primary metabolites or biomarkers of exposure. These six time series plots compare bedroom and kitchen concentrations, showing clear elevations when residents were in the bedroom overnight. The kitchen was chosen for comparison, as it is the most closely coupled indoor sampling location. Exceptions to the general trends may result from persistent emissions related to kitchen activity throughout the day or from changes in ventilation of the bedroom, such as the opening of the bedroom door at night, allowing the transport of emissions originating from sources in the bedroom to other rooms in the house.

**Figure 3:**
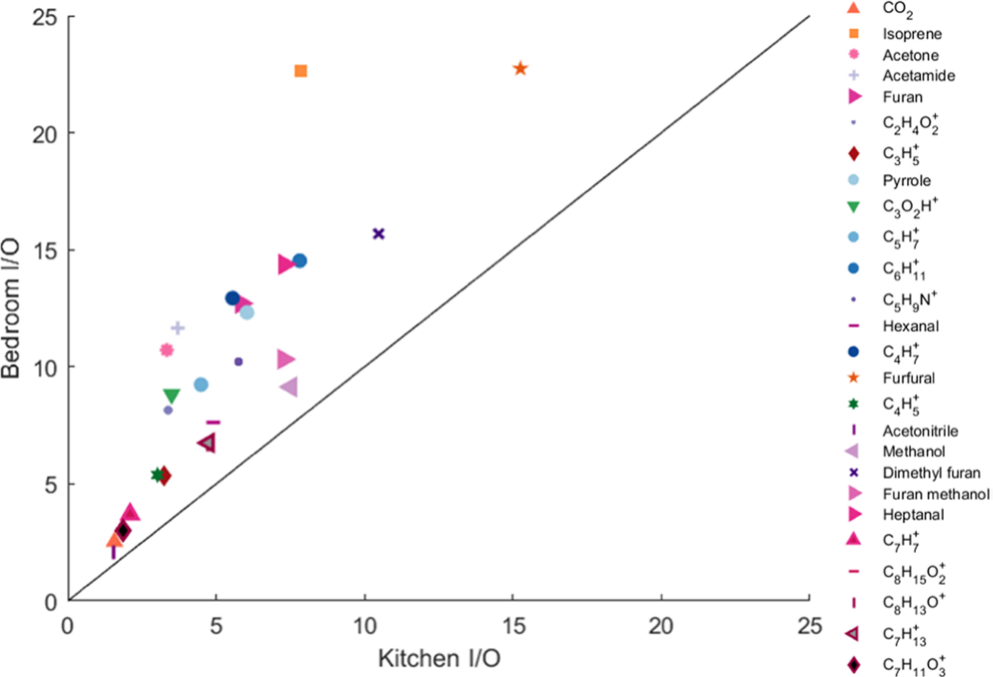
Average overnight indoor/outdoor (I/O) ratios in the bedroom and kitchen of compounds that are well-correlated (*R* ≥ 0.70) with CO_2_. Each compound is color-coded according to the legend on the right. All compounds are above the 1:1 line, indicating higher emissions from sources in the bedroom at night.

To ensure that the elevated overnight concentrations resulted from an indoor source in the bedroom, the indoor/outdoor ratios (I/O) in the kitchen (*x*-axis) and bedroom (*y*-axis) for all 25 compounds that correlated well with CO_2_ (and CO_2_ itself) are compared in Figure 3. An I/O was computed for the 3 h peak period for each of the 12 analyzed nights, with the averages of those 12 determinations reported in the figure. Bedroom I/O ratios are reported for quantified VOC concentrations in Table S4. The 26 compounds displayed in Figure 3 had higher concentrations indoors than outdoors, given that all average I/O values are above 1 in both rooms; this result confirms that they are largely of indoor origin, which is consistent with previous work.^13^ Additionally, every compound had higher average I/O values in the bedroom at night than in the kitchen, indicated by the fact that they are all above the 1:1 line in the figure. This outcome is expected given the results of Figure 1 but highlights the exclusivity of (a) compounds elevated in the kitchen versus (b) compounds that show a good correlation with CO_2_ in the bedroom. The observation offers further evidence that their enhancements in bedroom air are caused by sources in the bedroom rather than by transport from one room to another. Some compounds that showed significant enhancement in the bedroom and correlated well with CO_2_, such as isoprene and acetone, are known human bioeffluents and are explored further in the SI. While many VOCs have multiple sources and emission pathways, it is possible that human emissions can make a substantial contribution to overall VOC enhancements measured in bedroom air in this study. Nevertheless, the wide variety of possible sources, formation pathways, and emission pathways makes it difficult to offer conclusive evidence of why certain compounds were found to be elevated in the bedroom at night.

### H3 Bedroom Air Change Rates and Resulting Enhanced VOC Emission Rates

Air change rates for each of the 12 nights, shown in Table 1, were calculated using measured CO_2_ concentrations from the onset of the sleeping period to the time of peak concentration in the bedroom at H3 by applying its human emission factors that have been previously established experimentally and shown to vary by age, gender, activity, weight, and other factors.^29−31,38,45−47^ Here, estimates for one male occupant (ER_CO_2__ = 27 g h^–1^) and one female occupant (ER_CO_2__ = 21 g h^–1^) during sleep^47^ were used to determine average bedroom ACR values for each of the 12 nights used in detailed analysis. The median lower (upper)-bound bedroom ACR over these 12 nights was 0.54 (0.72) h^–1^. Calculated ACRs with respect to the outdoors (and on about six nights with respect to the kitchen) are generally low (i.e., below 1.0 h^–1^) because doors and windows were closed and there was no mechanical ventilation. These findings are largely consistent (except for Nov 23 and 25) with bedroom ACRs reported by Bekö et al. for children’s bedrooms in Denmark.^48^ While there are no metadata to confirm that the doors and windows were closed, the overnight accumulation of CO_2_ and VOCs in the bedroom would suggest that there was limited air exchange between this room and the other indoor spaces on the 12 nights included in the analysis.

**Table 1:** Air Change Rate (ACR) in the Bedroom with One Male Occupant (ER_CO_2__ = 27 g h^–1^) and One Female Occupant (ER_CO_2__ = 21 g h^–1^), with Bounding Estimates that Assume Ventilation Is Either Solely from Outdoors (Lower Bound) or Solely from the Kitchen (Upper Bound)^a^

date	(ppm)	Δ*t* (h)	*T*_avg_ (°C)	ACR (h^–1^)^b^
Nov 18	1300	4.0	21.0	0.49–0.57
Nov 23	930	4.0	19.5	2.10–5.40
Nov 25	990	3.5	20.7	1.27–3.01
Nov 26	1570	4.0	20.5	0.65–0.89
Nov 27	1670	7.5	20.0	0.66–0.88
Nov 28	1580	7.0	20.5	0.66–0.95
Nov 30	1820	5.5	20.8	0.41–0.47
Dec 01	2100	4.0	20.5	0.39–0.47
Dec 02	2200	6.0	20.8	0.37–0.54
Dec 03	1870	4.0	21.5	0.27–0.36
Dec 05	1450	4.0	21.0	0.55–0.77
Dec 06	1290	3.5	20.5	0.52–0.68

a Average CO_2_ concentrations over the reported duration, from the beginning of the sleeping period to peak CO_2_, are also reported in ppm.

b ACR = 0.5 h^–1^ for the H3 bedroom (*V* = 37 m^3^) with two occupants is equal to a ventilation rate of 2.6 L s^–1^ person^–1^ (∼5 cfm person^–1^), which is one-third of the minimum ASHRAE Standard 62.2–2016 recommendation.^49^

Emission rates over the entire overnight occupied period for each of the 12 nights were calculated using eq 2 for about 60 VOCs under two limiting conditions and are reported in Table 2 in units of μg h^–1^. Most have high relative standard deviations, indicating a wide range of emission rates that could be at least partially due to variations in calculated air change rates. Some compounds do not show a significant change in emission rates for the bounding assumptions, while others are a factor of 2 or more higher under the upper-limit assumption that all ventilation air comes from outdoors. Notably, compounds like vanillin and D5 siloxane actually have a slightly higher average emission rate under the assumption that all ventilation air enters the bedroom from the kitchen. Some compounds, such as acetone, isoprene, and 6-MHO, have per-person emission factors that are reported in numerous studies and vary significantly (EF_acetone_ = 34–1160 μg h^–1^ person^–1^,^14,26,50,51^ EF_isoprene_ = 26–166 μg h^–1^ person^–1^,^14,50,51^ and EF_6MHO_ = 3–39 μg h^–1^ person^–1^^14,26,51^). On a per-person basis, EF_acetone_ and EF_6MHO_ from this study fall within the literature values, while EF_isoprene_ values in the present study are ∼4–5× higher, likely due to contributions from other sources besides occupants. Other compounds, such as hexanal and heptanal, do not have emission factors that are as widely reported and therefore show less variation (EF_hexanal_ = 1.4 μg h^–1^ person^–1^ and EF_heptanal_ = 0.5 μg h^–1^ person^–1^^26^). Emission factors from this study are also ∼4× higher than these reported values.

**Table 2:** Average Upper (Air Exchange with Outdoors) and Lower (Air Exchange with Kitchen) Bounds for a Subset of Identified VOC Species and Their Emission Rates (ER) in Units of μg h^–1^ in the Bedroom over 12 Nights at H3 Using the Full Occupancy Period

*m*/*z*	ion formula	name	ER_upper_ (mean ± SD)	ER_lower_ (mean ± SD)
49.016	CH_5_S^+^	methanethiol	2.0 ± 1.1	
59.049	C_3_H_7_O^+^	acetone	1530 ± 440	1120 ± 500
60.020	C_2_H_4_O_2_^+^	acetate	4.3 ± 1.8	2.8 ± 1.7
60.051	C_2_H_6_NO^+^	acetamide	48 ± 18	44 ± 20
68.051	C_4_H_6_N^+^	pyrrole	3.5 ± 1.2	2.3 ± 1.1
68.995	C_3_O_2_^+^	carbon suboxide	42.4 ± 0.9	1.8 ± 1.0
69.033	C_4_H_5_O^+^	furan	25 ± 9	11.0 ± 10.0
69.069	C_5_H_9_^+^	isoprene	1030 ± 330	890 ± 310
71.049	C_4_H_7_O^+^	MVK + isomers	63 ± 27	16 ± 44
71.085	C_5_H_11_^+^	pentene	18.6 ± 9.0	3.5 ± 8.9
77.033	C_3_H_9_S^+^	propanethiol	2.9 ± 1.8	1.40 ± 0.84
83.049	C_5_H_7_O^+^	furan methanol	21 ± 9	1.7 ± 9.6
85.028	C_4_H_5_O_2_^+^	furanone	7.9 ± 5.0	1.4 ± 6.4
85.064	C_5_H_9_O^+^	cyclopentanone + isomers	14.6 ± 8.3	6.4 ± 2.2
85.100	C_6_H_13_^+^	hexene	6.5 ± 4.4	2.5 ± 1.9
97.063	C_6_H_9_O^+^	dimethyl furan	22 ± 14	4.3 ± 5.4
101.026	C_4_H_5_O_3_^+^	succinic anhydride	3.2 ± 2.0	
101.060	C_5_H_9_O_2_^+^	4-OPA	18.7 ± 9.8	2.7 ± 6.4
101.096	C_6_H_13_O^+^	hexanal	13.4 ± 6.6	4.0 ± 2.7
103.057	C_4_H_7_O_3_^+^	acetate anhydrate	8.7 ± 4.7	0.8 ± 8.2
104.056	C_7_H_6_N^+^	benzonitrile	0.75 ± 0.51	0.30 ± 0.52
105.034	C_4_H_9_OS^+^	methional	3.1 ± 1.6	1.6 ± 2.1
107.050	C_7_H_7_O^+^	benzaldehyde	76 ± 44	56 ± 39
109.099	C_8_H_13_^+^	6-MHO fragment	17.9 ± 10.4	10.4 ± 7.6
115.112	C_7_H_15_O^+^	heptanal	8.1 ± 4.6	2.9 ± 1.4
117.089	C_6_H_13_O_2_^+^	hexanoic acid	7.7 ± 6.3	2.2 ± 2.4
121.066	C_8_H_9_O^+^	anisaldehyde	6.5 ± 3.9	3.2 ± 2.2
127.112	C_8_H_15_O^+^	6-MHO + others	58 ± 30	38 ± 25
136.022	C_7_H_6_NS^+^	benzothiazole	3.2 ± 1.8	3.5 ± 2.1
137.060	C_8_H_9_O_2_^+^	4-anisaldehyde	6.6 ± 3.4	3.6 ± 4.5
137.132	C_10_H_17_^+^	monoterpenes	470 ± 260	173 ± 178
139.078	C_8_H_11_O_2_^+^	creosol	2.0 ± 1.3	1.90 ± 1.50
141.128	C_9_H_17_O^+^	nonenal + others	1.70 ± 1.00	0.45 ± 1.40
143.145	C_9_H_19_O^+^	C9 saturated carbonyl	910 ± 530	540 ± 175
153.055	C_8_H_9_O_3_^+^	vanillin + others	8.3 ± 7.4	10.0 ± 13.5
157.159	C_10_H_21_O^+^	C10 saturated carbonyl	6.1 ± 3.3	4.3 ± 2.2
171.173	C_11_H_23_O^+^	C11 saturated carbonyl	1.10 ± 1.40	0.52 ± 1.10
371.102	C_10_H_31_O_5_Si_5_^+^	D5 siloxane	147 ± 129	187 ± 250

It is worthwhile to highlight the emission rates of compounds associated with human breath, such as isoprene and acetone, and of compounds associated with skin, such as 6-MHO (skin oil oxidation product) and D5 siloxane (component of personal care products). There is some overlap between enhanced bedroom VOCs and species that correlate well with CO_2_, suggesting a potential relationship with human emissions. However, because the direct sampling of human breath or skin emissions was not undertaken in this study, the contribution of occupants as a source category to emissions of these species cannot be isolated. Periods considered to be “vacant” with respect to occupants consisted of researchers entering the site to perform experiments and therefore are not truly vacant. Figure S4 shows diel profiles in three sampling locations (bedroom, kitchen, and outdoors) for CO_2_, isoprene, and acetone and reveals similar behaviors for all three species. Outdoor concentrations are low and are relatively stable, kitchen concentrations are higher and vary throughout the day, and bedroom concentrations show accumulation overnight but similar behavior to the kitchen during the day. This may be because the bedroom door was open; however, there is not enough information from the occupants’ logs or the metadata sensors to verify this inference. Figure S5 compares the *R* values of VOCs with respect to isoprene and CO_2_. (A similar plot for acetone was not included because fewer compounds showed a high correlation with this compound as compared to that of CO_2_.) The time series of O_3_ and its skin oil oxidation products (Figure S6) and the diurnal plot of D5 siloxane (Figure S7) do not show accumulation overnight; this feature is explored further in the SI. Bioeffluents can be used as markers of different human attributes, such as metabolism, food or beverage ingestion, and, to a certain extent, health status; however, caution is required as bioeffluents can be formed through different pathways, can be tied to multiple food or beverage sources, or can result from several activities or exposure pathways^24,43^ which adds to the difficulty of establishing emission thresholds for healthy or unhealthy individuals. For example, acetone is a biomarker of diabetes but can also be indicative of recent exercise.^43,49^ Similar conundrums have been explored for other VOCs.^49−52^

In this paper, we have characterized VOC composition in a normally occupied bedroom during sleep and compared it to overnight composition in the kitchen and outdoors.^53−55^ We have determined bedroom air change rates based on established CO_2_ emission factors from occupants. Combining time series VOC concentration data with air change rate information, we have estimated emission rates in the bedroom of VOCs whose overnight concentrations are enhanced. The data reveal that two-thirds of detected VOC concentrations are higher in the bedroom than in the kitchen overnight, with nearly 100 compounds meeting or exceeding an enhancement ratio threshold of 1.30. These enhanced VOC concentrations are indicative of indoor sources emitting specifically into the bedroom. This paper has also explored a subset of bedroom-enhanced VOCs known to be bioeffluents as associated with breath emissions and skin oil oxidation. Exposure to such species can potentially have implications for the health status and cognitive ability. Differences among their formation pathways led to differences in overnight temporal concentration patterns. The enhancement of D5 siloxane, as consistently observed in the morning, is most likely attributable to the application of personal care products after occupants wake up. Processes that affect species concentrations in bedrooms are relevant to public health, as the large proportion of time spent by populations in such microenvironments can contribute substantially to aggregate personal exposures to VOCs. Understanding indoor air composition and determining emission rates for compounds enhanced in bedroom air overnight can lead to more accurate modeling of indoor air chemistry and exposure, as well as an understanding of how indoor air, once transported to the outdoors, plays a role in atmospheric chemistry.

Approximately, one-third of an individual’s time indoors is spent in their bedroom, making this a microenvironment of high interest in terms of exposure, given its dependence on both concentration and duration. The accumulation of CO_2_ and VOCs in this microenvironment during the sleeping period every night is worth investigating because of the known effects of CO_2_ exposure on cognitive ability and sleep quality.^7,56,57^ There are also emerging studies showing that bioeffluents, a category of VOCs that can be elevated in bedrooms during sleep, also negatively impact sleep quality.^57^ This work provides a strong foundation for characterizing bedroom indoor air composition and identifying species to which humans are exposed for long durations at high concentrations, potentially affecting their sleep quality and, consequently, their overall health.

## Supporting Information Available

Observational campaign description and layout; calibration gas composition; CO_2_ measurement corrections; CO_2_ overnight enhancement; average bioeffluent concentrations at night; diel plots of breath emission tracers; *R*-value comparisons; ozone and oxidation product time series; and D5 siloxane diurnal profiles (PDF)
