# Supplementary material for: Bedroom Concentrations and Emissions of Volatile Organic Compounds during Sleep
Source: Environ Sci Technol. 2024 Apr 24;58(18):7958–67. doi: 10.1021/acs.est.3c10841 (PMC11080066; doi:10.1021/acs.est.3c10841)
Supplement: Supplementary file 1 — es3c10841_si_001.pdf [file es3c10841_si_001.pdf]

Supporting Information for

Bedroom Concentrations and Emissions of Volatile Organic Compounds During Sleep

Betty Molinier[1], Caleb Arata[2,3], Erin F. Katz[2], David M. Lunderberg[2,3], Jennifer Ofodile[3], Brett C. Singer[4], William W Nazaroff[1], Allen H. Goldstein[1,3]

[1]Department of Civil and Environmental Engineering, University of California, Berkeley, California 94720, United States, [2]Department of Chemistry, University of California, Berkeley, California 94720, United States, and [3]Department of Environmental Science, Policy and Management, University of California, Berkeley, California 94720, United States, [4]Indoor Environment Group and Residential Building Systems Group, Lawrence Berkeley National Laboratory, Berkeley, California 94720, USA.

SI Contains:

20 pages

5 tables

7 figures

SI Table of Contents

1. (*page S3*) - House 3 Layout
    a. **Figure S1.** Schematic of H3 layout and sampling locations
2. (*page S4*) - PTR-TOF-MS Calibration Gases
    a. **Table S1.** Standard Gas 1
    b. **Table S2.** Standard Gas 2
3. (*page S5*) - CO_2 Measurement Correction
    a. **Figure S2.** Scatter plot
    b. **Figure S3.** Time series
4. (*page S7*) - CO_2 Accumulation During Occupied Nights
    a. **Table S3.** CO_2 Enhancement Over Sleeping Periods
5. (*page S8*) - Nighttime VOC Concentrations at H3
    a. **Table S4.** Summary statistics on VOC signals in the bedroom at H3
6. (*page S13*) - Breath Emission Tracers
    a. **Figure S4.** Diel plots of CO_2, isoprene, and acetone at H3
    b. **Figure S5.** Scatter plots of VOC R values with respect to isoprene and CO_2
7. (*page S15*) - Bedroom VOC Emission Rates
    a. **Table S5.** Average upper and lower bounds for bedroom VOC emission rates
8. (*page S17*) - Skin Emission Signals
    a. **Figure S6.** Ozone and ozone oxidation product time series
    b. **Figure S7.** D5 siloxane diurnal pattern in H3 bedroom
9. (*page S20*) - References

House 3 Layout

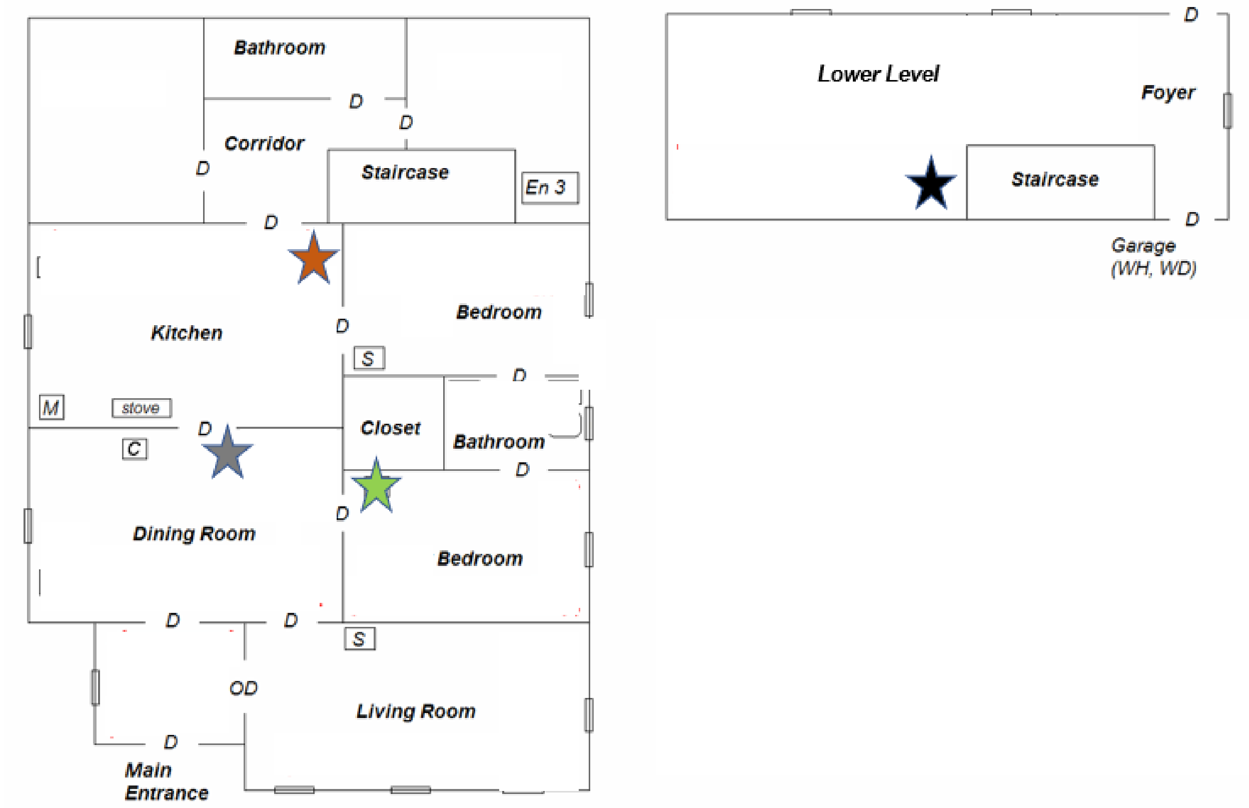

Figure S1. House 3 (H3) layout. Orange, green, and black stars indicate sampling line inlet locations, and the gray star indicates a tracer release location. Additional sampling locations not shown are in the attic (above main floor), garage (same elevation as lower level), and outside. Additional tracer release locations not shown are in the attic and garage.

Instruments were situated in the basement/garage area of the H3 study site to minimize interference with the occupants' daily lives. Researchers were able to maintain and retrieve data from the instruments without entering the house and disrupting the normal pattern of occupancy. Air sampling lines and in-situ low-cost sensors were placed inside the residence as indicated in Figure S1.

PTR-TOF-MS Calibration Gases

Table S1. Composition of Standard Gas 1 used to calibrate the PTR-TOF-MS at H3.

Ion mass (*m/z*)	Ion formula	Name	Concentration (ppb)
33.033	CH_5O^+	Methanol	543
42.034	$C_2H_3NH^+$	Acetonitrile	512
45.033	$C_2H_5O^+$	Acetaldehyde	429
47.049	$C_2H_7O^+$	Ethanol	524
57.033	$C_3H_5O^+$	Acrolein	497
63.026	$C_2H_7S^+$	Dimethyl sulfide	495
69.070	$C_5H_9^+$	Isoprene	475
71.049	$C_4H_7O^+$	Methacrolein	493
79.054	$C_6H_7^+$	Benzene	501
93.070	$C_7H_9^+$	Toluene	475
107.086	$C_8H_{11}^+$	p-Xylene	480
109.065	$C_7H_9O^+$	p-Cresol	497
121.101	$C_9H_{13}^+$	1,3,5-Trimethylbenzene	475
223.064	$C_6H_{19}O_3Si_3^+$	D3 siloxane	496
445.120	$C_{12}H_{37}O_6Si_6^+$	D6 siloxane	485

Table S2. Composition of Standard Gas 2 used to calibrate the PTR-TOF-MS at H3.

Ion mass (*m/z*)	Ion formula	Name	Concentration (ppb)
33.033	CH_5O^+	Methanol	505
47.049	$C_2H_7O^+$	Ethanol	509
59.049	$C_3H_7O^+$	Acetone	483
61.065	$C_3H_9O^+$	Propanol	486
69.033	$C_4H_5O^+$	Furan	505
75.080	$C_4H_{11}O^+$	Butanol	503
97.028	$C_5H_5O_2^+$	Furfural	499
107.049	$C_7H_7O^+$	Benzaldehyde	517
137.133	$C_{10}H_{17}^+$	Monoterpenes	480.8
127.112	$C_8H_{15}O^+$	6-MHO	472
143.143	$C_9H_{19}O^+$	Nonanal	509
297.082	$C_8H_{25}O_4Si_4^+$	D4 siloxane	487
371.101	$C_{10}H_{31}O_5Si_5^+$	D5 siloxane	470

CO_2 Measurement Correction

The LICOR LI-820 instrument used to measure CO_2 in the sampling manifold did not record measurements higher than 2000 ppm. The Netatmo CO_2 sensors measured a broader range, including levels above 3000 ppm. Data from the Netatmo sensors, which were placed around the house, were used for analysis in this paper. A correction factor is applied to improve the accuracy of the Netatmo measurements to match the calibrated LICOR instrument. For the main bedroom, the data collected by the LICOR instrument from November 17 through December 06, 2021, was compared to Netatmo data, averaged over matching 30-minute sample periods.

To ensure that the LICOR measurements near 2000 ppm also did not interfere with the analysis, any LICOR measurements over 1800 ppm and Netatmo measurements over 2000 ppm were removed for determining the CO_2 measurement correction. The 'linortfit' function in MATLAB was applied to determine the best fit and correlation, as shown in Figure S2.

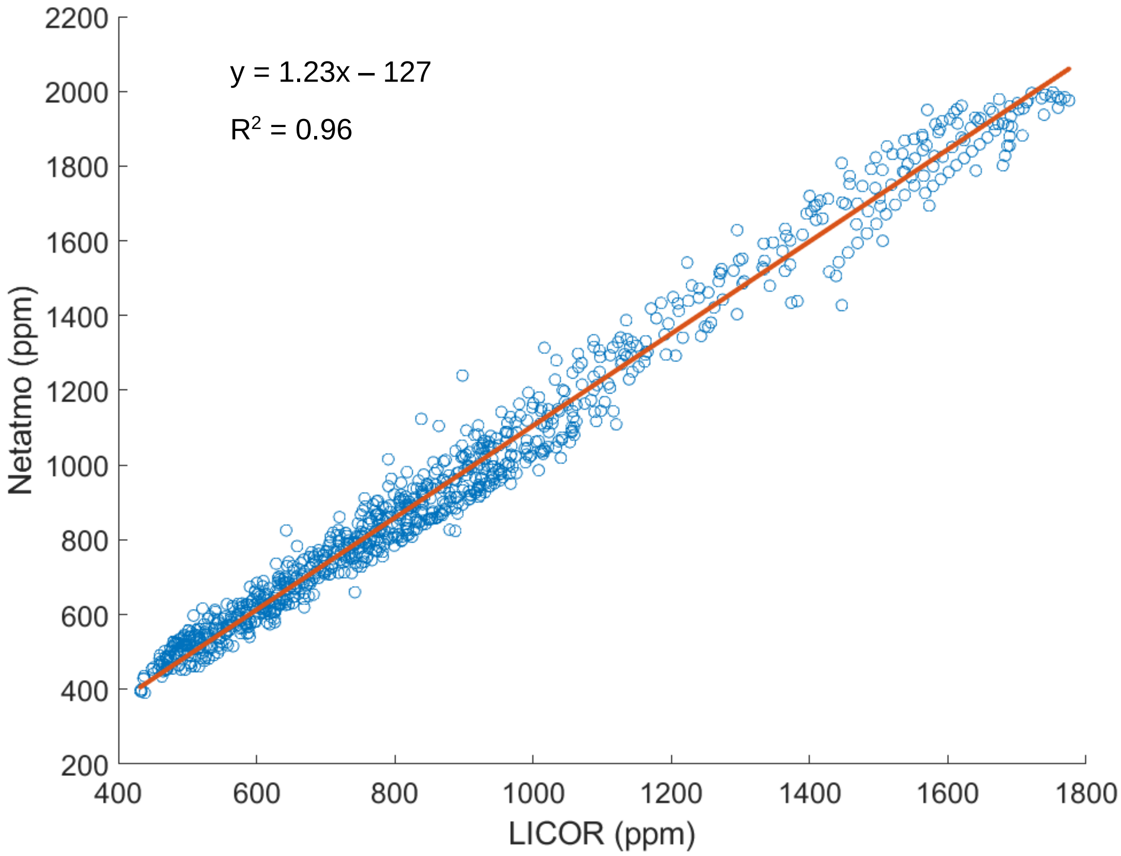

Figure S2. Scatter plot (blue) of bedroom CO_2 measurements (ppm) made by LICOR (*x*-axis) and Netatmo (*y*-axis) sensors with orthogonal least squares best fit line (orange). The equation for line of best fit is also provided.

Figure S3 compares the time series of the original Netatmo data, the LICOR data, and the corrected Netatmo data in the main bedroom over one of the last three weeks of the H3 campaign. The corrected Netatmo data (green) is used for all analyses reported in this paper.

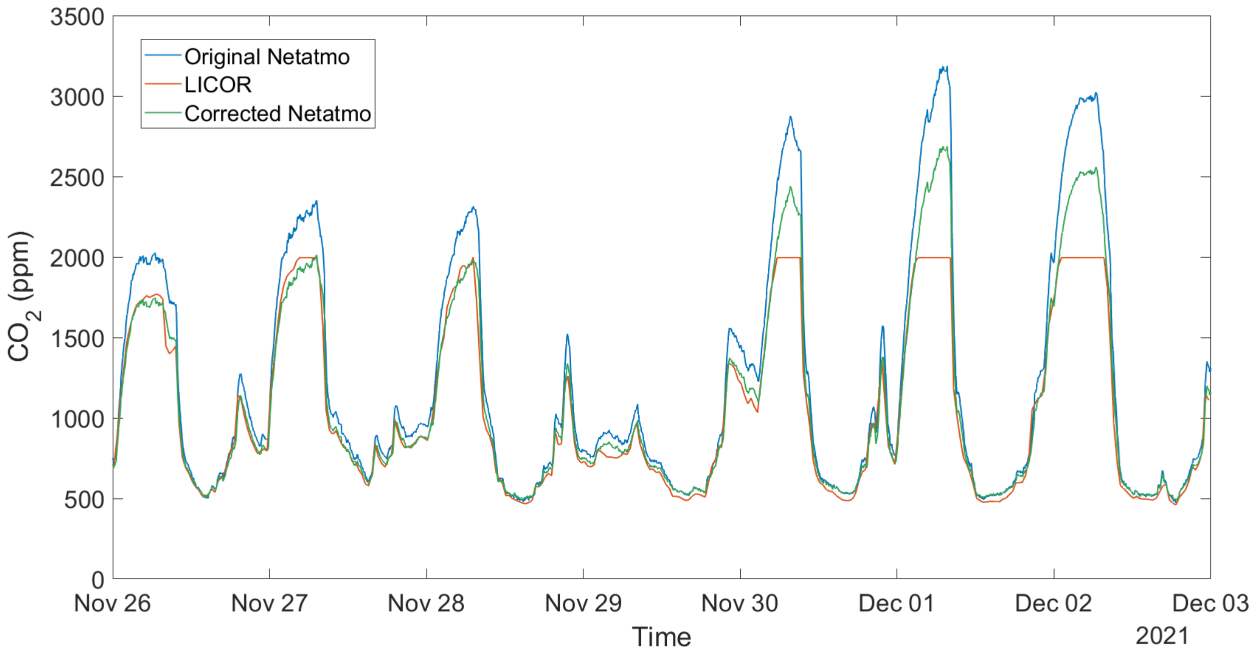

Figure S3. Time series of bedroom CO_2 concentrations over a week of the H3 campaign. The blue trace corresponds to the original Netatmo data, the orange trace corresponds to LICOR data, and the green trace corresponds to the corrected Netatmo data.

CO_2 Accumulation During Occupied Nights

There are a total of 18 days and nights with PTR-TOF-MS measurements in the main bedroom while occupants were present (and two additional vacant nights), but the data do not exhibit consistent behavior every night. The fifth-percentile CO_2 concentration over H3 was subtracted from the overall concentration profile to remove the background CO_2 baseline in the bedroom. To identify the nights that are indicative of significant emissions accumulating in the bedroom, the area under the background-subtracted CO_2 curve for each night was integrated using the 'trapz' function in MATLAB to determine whether an ad hoc substantial accumulation threshold of 1000 ppm-h was exceeded. Table S3 summarizes these findings, presenting the start times, duration of each analysis period, and the CO_2 accumulation for each relevant date. The analysis period includes the times during which occupants were in the bedroom as well as the period of decay in CO_2 concentration after occupants leave the room.

Table S3. Duration and CO_2 accumulation in the monitored bedroom on relevant nights during H3 sampling campaign.

**Date**	**Start Time**	**Duration (hours)**	**Accumulation CO_2 (ppm-h)**
Nov 17	00:00	11.0	**540**
Nov 18	00:00	12.0	4600
Nov 19	23:05†	8.5	**-11**
Nov 22	02:00	8.5	**590**
Nov 23	23:15†	13.0	2400
Nov 24	23:30†	9.0	**160**
Nov 25	00:15	8.0	2100
Nov 26	00:30	10.5	7900
Nov 27	23:15†	13.0	8400
Nov 28	00:15	10.0	5900
Nov 29	01:15	8.5	**890**
Nov 30	01:15	9.5	5500
Dec 01	23:43†	13.5	12000
Dec 02	23:50†	10.5	3800
Dec 03	00:30	12.0	2800
Dec 04	00:00	9.5	**390**
Dec 05	23:40†	10.0	6400
Dec 06	00:00	11.5	2600

Bolded values indicate those with CO_2 accumulation < 1000 ppm-h overnight.
†Indicates time from the night before if the occupants went to bed before midnight.

Based on the 1000 ppm-h selection criterion, the following nights were removed from further analysis: Nov 17, 19, 22, 24, 29, and Dec 04. The small negative accumulation value reported for Nov 19 may be a result of the manipulation experiments conducted by researchers while regular occupants were away. The remaining 12 nights, in which occupant bioeffluents materially affected the bedroom CO_2 levels overnight, were used in this paper for analysis of VOC composition, concentrations, and emissions in the occupied bedroom. Here, the duration includes the decay period of the CO_2 concentration profiles until the return to steady state, which continued after occupants left the bedroom. The duration used for air-change rate calculations was for the period

of increasing CO_2 concentrations only, and the duration used in emission rate calculations was for the time periods that occupants were in the bedroom only.

Nighttime VOC Concentrations at H3

Table S4 presents summary statistics on three-hour periods of peak concentrations of compounds at three sampling locations over the twelve occupied nights prescribed by the filtering techniques discussed in the previous section using the sleeping period durations listed in Table S3. We removed 25 compouThe ratio of the average 3-h peak bedroom and average 3-h peak kitchen concentrations is also reported. Values meeting the threshold C_{bed}/C_{kitch} ≥ 1.30 are indicated in bold. Data interpolated over the PTR-TOF-MS calibration periods (01:00-04:00 every morning) were included. We report 214 of the 239 detected VOC signals to focus on compounds not related to water clusters, the primary ion, or with a mass below m/z = 33.033 (methanol). Of the 214 signals, 94 showed an enhancement ratio of 1.30 or higher. For all those compounds, the corresponding outdoor concentration was either too low to be reported or resulted in an indoor-to-outdoor ratio of ≥ 3.0 (with one exception).

Table S4. Mean VOC concentrations (ppb) in the bedroom, kitchen, and outdoors during twelve selected occupied nights.[a]

Ion mass *(m/z)*	Ion formula	Name	Occupied Night Averages, Peak 3 h (ppb; n = 12)				
			C_{bed}	C_{kitch}	C_{bed}/C_{kitch}	C_{out}	C_{bed}/C_{out}
33.033	CH_5O^+	methanol	97	87	1.12	10.6	9.1
41.038	$C_3H_5^+$	alkyl fragment	2.6	1.62	**1.61**	0.49	5.3
42.009			0.115	0.179	0.64	0.028	4.1
42.034	$C_2H_4N^+$	acetonitrile	0.25	0.20	1.26	0.122	2.1
43.054	$C_3H_7^+$	propene/propanol	10.2	8.7	1.17	3.1	3.3
43.997			0.108	0.165	0.65	0.21	0.53
45.033	$C_2H_5O^+$	acetaldehyde	7.6	7.2	1.06	0.78	9.8
45.993			0.109	0.25	0.43	0.088	1.23
46.030			0.40	0.33	1.23	0.049	8.2
46.073	Tracer	propene-D6	0.023	0.018	1.23	ND	ND
47.013	$CH_3O_2^+$	formic acid	4.6	4.6	0.99	ND	ND
47.023	$H_3N_2O^+$		2.3	2.2	1.08	ND	ND
47.049	$C_2H_7O^+$	ethanol	230	210	1.09	18.5	12.3
48.001			0.044	0.043	1.01	0.016	2.7
48.016	$H_2O_2N^+$	nitrous acid	0.094	0.102	0.93	0.007	13.5
48.085			0.028	0.026	1.12	ND	ND
49.016	CH_5S^+	methanethiol	0.083	0.061	**1.37**	0.011	7.8
49.028	$CH_5O_2^+$	methane diol	0.069	0.076	0.91	ND	360
50.001			0.010	0.047	0.22	0.074	0.14
51.007			0.038	0.026	**1.48**	0.007	5.7
51.995	NH_3Cl^+	chloramine	0.010	0.018	0.56	ND	ND
53.038	$C_4H_5^+$	alkyl fragment	0.058	0.032	**1.82**	0.011	5.4
54.033	$C_3H_4N^+$	acrylonitrile	0.037	0.033	1.09	0.009	3.9
55.039			2.3	0.71	**3.2**	ND	ND
55.053	$C_4H_7^+$		2.1	1.12	**1.91**	0.165	12.9
57.033	$C_3H_5O^+$	acrolein	2.7	2.2	1.27	0.25	11.2
57.070	$C_4H_9^+$	butene	4.8	4.4	1.09	1.18	4.1
59.049	$C_3H_7O^+$	acetone	39	11.5	**3.4**	3.6	10.7
60.020	$C_2H_4O_2^+$		0.141	0.063	**2.2**	0.017	8.1

60.051	$C_2H_6NO^+$	acetamide	1.54	0.48	**3.2**	0.132	11.7
60.088	Tracer	butene-D3	0.33	0.42	0.80	0.023	14.4
61.029	$C_2H_5O_2^+$	acetic acid	34	30	1.13	ND	ND
62.028			1.78	2.5	0.70	ND	ND
64.030			0.018	0.011	**1.58**	ND	ND
65.024	$CH_5O_3^+$		0.133	0.091	**1.46**	ND	ND
65.034	$C_5H_5^+$		0.070	0.045	**1.58**	ND	ND
65.086			0.051	0.033	**1.57**	0.005	10.2
67.053	$C_5H_7^+$	alkyl fragment	0.197	0.094	**2.1**	0.021	9.2
68.051	$C_4H_6N^+$	pyrrole	0.095	0.038	**2.5**	0.008	12.3
68.995	$C_3O_2^+$		0.072	0.030	**2.4**	0.008	8.8
69.033	$C_4H_5O^+$	furan	0.65	0.29	**2.2**	0.051	12.7
69.069	$C_5H_9^+$	isoprene	28	8.2	**3.4**	1.23	23
70.036			0.032	0.020	**1.59**	0.008	4.2
71.049	$C_4H_7O^+$	MVK + isomers	1.62	1.04	**1.57**	0.177	9.1
71.085	$C_5H_{11}^+$	pentene	0.56	0.40	**1.41**	0.133	4.2
73.029	$C_3H_5O_2^+$	acrylic acid	0.23	0.21	1.11	0.017	13.3
73.043			0.23	0.22	1.07	0.042	5.5
73.064	$C_4H_9O^+$	MEK + isomers	0.90	0.83	1.08	0.25	3.6
74.032			0.028	0.017	**1.62**	ND	ND
74.059	$C_3H_8NO^+$		ND	ND	ND	ND	ND
75.025			0.24	0.190	1.29	ND	ND
75.043	$C_3H_7O_2^+$	propionic acid	0.81	0.65	1.24	0.095	8.5
76.024			0.019	0.014	**1.34**	ND	ND
77.009			0.013	ND	ND	ND	ND
77.033	$C_3H_9S^+$	propanethiol	0.068	0.043	**1.56**	0.010	6.4
78.997	$C_2H_4OCl^+$		0.020	0.016	1.24	0.005	4.1
79.016	$C_2H_7OS^+$	2-thioethanol	0.039	0.033	1.19	0.013	2.9
79.052	$C_6H_7^+$	benzene	0.55	0.45	1.22	0.28	1.93
80.040			0.038	0.013	**3.0**	ND	ND
80.990	$C_2H_3FCl^+$		0.076	0.071	1.08	0.041	1.85
81.036	$C_5H_5O^+$		0.34	0.192	**1.76**	ND	ND
82.945	$CHCl_2^+$		1.32	1.78	0.75	0.32	4.1
83.049	$C_5H_7O^+$	furan methanol	0.45	0.27	**1.63**	0.043	10.3
83.085	$C_6H_{11}^+$	alkyl fragment	2.3	1.16	**1.96**	0.157	14.5
84.051	$C_4H_5NO^+$		0.033	0.017	**1.90**	ND	ND
84.088	$C_5H_9N^+$		0.170	0.092	**1.85**	0.017	10.2
85.028	$C_4H_5O_2^+$	furanone	0.151	0.106	**1.42**	0.017	8.7
85.064	$C_5H_9O^+$	cyclopentanone + isomers	0.31	0.186	**1.65**	0.027	11.2
85.100	$C_6H_{13}^+$	hexene	0.179	0.137	**1.31**	0.059	3.1
86.061	$C_4H_8NO^+$		0.021	0.009	**2.2**	ND	ND
86.939	Cl_2OH^+		0.096	0.118	0.81	0.024	4.0
87.044	$C_4H_7O_2^+$	diacetyl?	0.43	0.33	**1.32**	0.040	10.9
87.079	$C_5H_{11}O^+$	pentanal	0.31	0.25	1.25	0.046	6.7
88.044	C4H8S$^+$		0.029	0.012	**2.4**	ND	ND
88.075	$C_4H_{10}NO^+$		0.027	0.017	**1.54**	ND	ND
89.043	$C_3H_5O_3^+$		0.27	0.098	**2.7**	ND	ND
89.058	$C_4H_9O_2^+$	butyric acid	0.37	0.32	1.15	0.044	8.5
90.047			0.017	0.007	**2.5**	ND	ND
91.053	$C_7H_7^+$		0.31	0.173	**1.80**	0.084	3.7
92.059	$C_7H_8^+$		0.050	0.041	1.22	0.020	2.5
93.039	$C_6H_5O^+$	aromatic fragment?	0.185	0.138	**1.34**	0.037	5.0
93.069	$C_7H_9^+$	toluene	1.00	0.89	1.12	0.53	1.88

95.034	$C_6H_7O^+$	phenol	0.038	ND	ND	ND	ND
95.083	$C_7H_{11}^+$		0.32	0.26	1.25	0.040	8.0
97.028	$C_5H_5O_2^+$	furfural	1.54	1.20	1.28	0.068	23
97.063	$C_6H_9O^+$	dimethyl furan	0.35	0.26	**1.34**	0.022	15.7
97.100	$C_7H_{13}^+$		0.43	0.30	**1.44**	0.063	6.8
98.958			0.019	0.017	1.14	0.013	1.42
99.008	$C_4H_3O_3^+$		0.015	0.015	0.99	ND	ND
99.044	$C_5H_7O_2^+$	furfuranol	0.062	0.052	1.19	0.010	6.4
99.080	$C_6H_{11}O^+$	cis-3-hexenal + others	0.170	0.138	1.23	0.020	8.5
101.026	$C_4H_5O_3^+$	succinic anhydride	0.062	0.047	**1.33**	0.012	5.4
101.060	$C_5H_9O_2^+$	4-OPA	0.30	0.21	**1.42**	0.021	14.0
101.096	$C_6H_{13}O^+$	hexanal	0.25	0.157	**1.56**	0.032	7.6
102.061			0.019	ND	ND	ND	ND
102.091			0.038	0.016	**2.4**	ND	ND
102.936			ND	ND	ND	ND	ND
103.057	$C_4H_7O_3^+$	acetate anhydrate	0.069	0.037	**1.89**	ND	ND
104.056	$C_7H_6N^+$	benzonitrile	0.015	0.011	**1.41**	ND	ND
105.034	$C_4H_9OS^+$	methional	0.053	0.038	**1.38**	0.010	5.3
105.068	$C_8H_9^+$	styrene	0.164	0.156	1.05	0.066	2.5
107.050	$C_7H_7O^+$	benzaldehyde	1.28	0.64	**2.0**	0.129	9.9
107.084	$C_8H_{11}^+$		0.80	0.72	1.11	0.47	1.71
108.022			0.027	0.022	1.21	0.005	4.9
108.087	$C_7H_{10}N^+$	dimethylpyridine	0.064	0.056	1.15	0.034	1.91
108.989			0.084	0.068	1.23	0.048	1.76
109.028	$C_6H_5O_2^+$		0.055	0.050	1.10	0.017	3.2
109.064	$C_7H_9O^+$	cresol	0.63	0.49	1.29	0.126	5.0
109.099	$C_8H_{13}^+$	6-MHO fragment?	0.27	0.165	**1.64**	0.026	10.6
109.993			0.009	0.010	0.97	0.008	1.15
110.066			0.015	0.011	**1.36**	0.005	2.8
111.044	$C_6H_7O_2^+$	benzenediol	0.069	0.059	1.16	0.011	6.4
111.081	$C_7H_{11}O^+$		0.152	0.107	**1.43**	0.020	7.6
111.116	$C_8H_{15}^+$	1-octen-3-ol frag + others	0.25	0.165	**1.51**	0.033	7.4
113.024	$C_5H_5O_3^+$		0.011	0.008	1.26	ND	ND
113.046			0.011	0.008	**1.36**	ND	ND
113.059	$C_6H_9O_2^+$		0.036	0.028	1.29	0.007	4.9
113.096	$C_7H_{13}O^+$		0.074	0.052	**1.43**	0.011	6.6
115.039	$C_5H_7O_3^+$		0.016	0.014	1.22	0.005	3.5
115.076	$C_6H_{11}O_2^+$		0.074	0.054	**1.37**	0.016	4.7
115.112	$C_7H_{15}O^+$	heptanal + others	0.115	0.071	**1.63**	0.008	14.4
116.907	CCl_3^+	chloroform	0.085	0.090	0.94	0.079	1.07
117.089	$C_6H_{13}O_2^+$	hexanoic acid	0.097	0.071	**1.37**	0.007	14.6
119.049			0.044	0.023	**1.96**	ND	12.0
119.083	$C_9H_{11}^+$		0.22	0.099	**2.3**	0.029	7.8
121.029	$C_7H_5O_2^+$		0.039	0.026	**1.51**	0.011	3.5
121.066	$C_8H_9O^+$	anisaldehyde	0.127	0.091	**1.39**	0.041	3.1
121.100	$C_9H_{13}^+$		0.45	0.36	1.25	0.26	1.73
122.002			0.009	0.005	**1.93**	0.005	1.83
123.044	$C_7H_7O_2^+$	benzoic acid	0.023	0.018	1.27	ND	ND
123.081			0.027	0.018	**1.48**	ND	ND
123.114	$C_9H_{15}^+$		0.046	0.038	1.21	0.014	3.3
125.024	$C_6H_5O_3^+$		0.016	0.016	1.02	0.006	2.5
125.06	$C_7H_9O_2^+$		0.026	0.014	**1.83**	ND	ND
125.096	$C_8H_{13}O^+$		0.081	0.050	**1.61**	0.012	6.7

125.132	$C_9H_{17}^+$		0.115	0.071	**1.63**	0.008	14.1
127.001			0.052	0.035	**1.48**	0.027	1.93
127.039	$C_6H_7O_3^+$		0.023	0.025	0.93	0.005	4.2
127.077	$C_7H_{11}O_2^+$		0.039	0.026	**1.53**	0.006	6.1
127.112	$C_8H_{15}O^+$	6-MHO + others	0.79	0.47	**1.70**	0.108	7.3
128.116			0.057	0.066	0.87	0.020	2.9
128.998			0.035	0.044	0.80	0.015	2.4
129.020			0.046	0.058	0.81	0.017	2.8
129.034			0.039	0.048	0.80	0.015	2.5
129.068	$C_6H_9O_3^+$		0.037	0.037	1.01	0.017	2.1
129.091	$C_7H_{13}O_2^+$		0.035	0.030	1.14	0.013	2.6
129.128	$C_8H_{17}O^+$		0.066	0.042	**1.56**	ND	ND
130.992			0.25	0.24	1.05	0.23	1.09
131.995			0.009	0.007	1.25	0.007	1.28
133.011	$C_8H_5O_2^+$		0.014	0.021	0.68	ND	ND
133.064	$C_9H_9O^+$	cinnamaldehyde	0.027	0.029	0.93	0.014	1.97
133.099	$C_{10}H_{13}^+$		0.036	0.032	1.10	0.017	2.1
135.082	$C_9H_{11}O^+$		0.052	0.042	1.25	0.014	3.8
135.115	$C_{10}H_{15}^+$	1,4-diethylbenzene	0.098	0.091	1.08	0.060	1.63
136.022	$C_7H_6NS^+$	benzothiazole	0.040	0.014	**3.0**	0.008	5.3
137.060	$C_8H_9O_2^+$	4-anisaldehyde	0.085	0.062	**1.36**	0.017	4.9
137.132	$C_{10}H_{17}^+$	monoterpenes	5.9	4.4	**1.34**	1.06	5.5
139.078	$C_8H_{11}O_2^+$	creosol	0.026	0.015	**1.78**	ND	ND
139.111	$C_9H_{15}O^+$		0.068	0.042	**1.62**	0.006	12.2
139.149	$C_{10}H_{19}^+$		0.033	0.023	**1.45**	0.007	4.8
141.092	$C_8H_{13}O_2^+$		0.016	0.014	1.16	ND	ND
141.128	$C_9H_{17}O^+$	nonenal + others	0.023	0.016	**1.44**	ND	ND
143.088	$C_7H_{11}O_3^+$		0.046	0.029	**1.58**	0.015	3.0
143.110	$C_8H_{15}O_2^+$		0.058	0.029	**2.0**	ND	ND
143.145	$C_9H_{19}O^+$	C9 saturated carbonyl	9.2	4.4	**2.1**	ND	ND
145.120	$C_8H_{17}O_2^+$	octanoic acid	0.020	0.019	1.05	ND	ND
146.976	$C_6H_5Cl_2^+$		0.017	0.018	0.93	0.016	1.02
147.063	$C_9H_7O_2^+$		0.074	0.091	0.82	0.023	3.3
147.996			0.005	ND	ND	ND	ND
148.063			0.017	0.020	0.84	0.006	3.0
148.974			0.043	0.057	0.75	0.021	2.0
149.026			0.23	0.29	0.78	0.065	3.4
149.040			0.54	0.70	0.78	0.171	3.2
149.094	$C_{10}H_{13}O^+$		0.158	0.182	0.87	0.027	5.8
149.991			0.030	0.032	0.92	0.022	1.31
150.041			0.112	0.147	0.77	0.040	2.8
150.994	$C_3F_6H^+$		0.017	0.023	0.73	ND	ND
151.035	$C_8H_7O_3^+$		0.082	0.101	0.81	0.023	3.5
151.111	$C_{10}H_{15}O^+$		0.076	0.074	1.03	0.021	3.6
152.034			0.012	0.015	0.76	ND	ND
153.015			0.019	0.012	**1.62**	ND	ND
153.055	$C_8H_9O_3^+$	vanillin + others	0.089	0.045	**2.0**	0.020	4.5
153.125	$C_{10}H_{17}O^+$	citral + others	0.049	0.040	1.24	0.021	2.4
155.088	$C_9H_{15}S$+	pentylthiopene	0.014	0.013	1.12	ND	ND
155.110	$C_9H_{15}O_2^+$		0.026	0.019	**1.36**	ND	ND
155.143	$C_{10}H_{19}O^+$	monoterpene alcohols	0.090	0.075	1.20	0.009	9.8
157.105	$C_8H_{13}O_3^+$		0.011	0.005	**2.0**	ND	ND
157.117	$C_9H_{17}O_2^+$		0.015	0.008	**1.79**	ND	ND

157.159	$C_{10}H_{21}O^+$	C10 saturated carbonyl	0.067	0.034	**1.97**	0.007	8.9
158.986			0.015	0.015	1.06	0.012	1.32
159.079	$C_8H_{15}O_3^+$		ND	0.005	ND	ND	ND
159.128	$C_8H_{19}O_2^+$		0.017	0.011	**1.50**	ND	ND
160.995	$C_7H_4ClF_2^+$	parachlorobenzotrifluoride	0.091	0.096	0.95	0.071	1.29
163.097	$C_{10}H_{11}O_2^+$		0.112	0.146	0.77	0.033	3.4
164.098			0.020	0.023	0.87	ND	ND
165.087	$C_{10}H_{13}O_2^+$		0.025	0.027	0.95	ND	ND
165.162	$C_{12}H_{21}^+$		0.012	0.014	0.83	0.006	1.96
167.060	$C_9H_{11}O_3^+$	veratraldehyde	0.089	0.108	0.82	0.024	3.7
168.060			0.015	0.020	0.76	0.005	3.3
169.051	$C_9H_{13}O_3^+$		0.012	0.016	0.75	ND	ND
171.173	$C_{11}H_{23}O^+$	C11 saturated carbonyl	0.012	0.008	**1.49**	0.005	2.7
177.160	$C_{13}H_{21}^+$		0.012	0.009	**1.30**	0.006	1.93
180.989			0.017	0.018	0.95	0.021	0.79
203.175	$C_{15}H_{23}^+$	sesquiterpenes	0.008	0.010	0.80	ND	ND
205.193	$C_{15}H_{25}^+$		0.031	0.030	1.04	ND	ND
207.028			0.020	0.017	1.17	ND	ND
221.151	$C_{14}H_{21}O_2+$	chromanol + others	0.008	0.009	0.97	ND	ND
223.063	$C_6H_{19}O_3Si_3^+$	D3 siloxane	0.064	0.051	1.24	0.012	5.3
245.224	$C_{18}H_{29}+$		0.006	0.006	1.01	0.005	1.43
281.050			0.044	0.041	1.06	0.015	2.9
282.049			0.011	0.012	0.86	ND	ND
297.086	$C_8H_{25}O_4Si_4^+$	D4 siloxane	0.040	0.041	0.98	0.007	5.5
371.102	$C_{10}H_{31}O_5Si_5^+$	D5 siloxane	0.72	0.36	**1.97**	0.22	3.2
445.120	$C_{12}H_{37}O_6Si_6^+$	D6 siloxane	0.20	0.21	0.95	0.193	1.04

[a] Each concentration entry represents the average value of 12 nighttime determinations in which each night is represented by the 3-h period producing the highest average bedroom concentration. Kitchen and outdoor average values were computed for the same time periods as the 3-h peaks in the bedroom. Bolded values indicate a $C_{\text{bed}}/C_{\text{kitchen}} \geq 1.30$. ND indicates "not determined," which reflects an average concentration value below the reporting limit of ~5 ppt.

Breath Emission Tracers

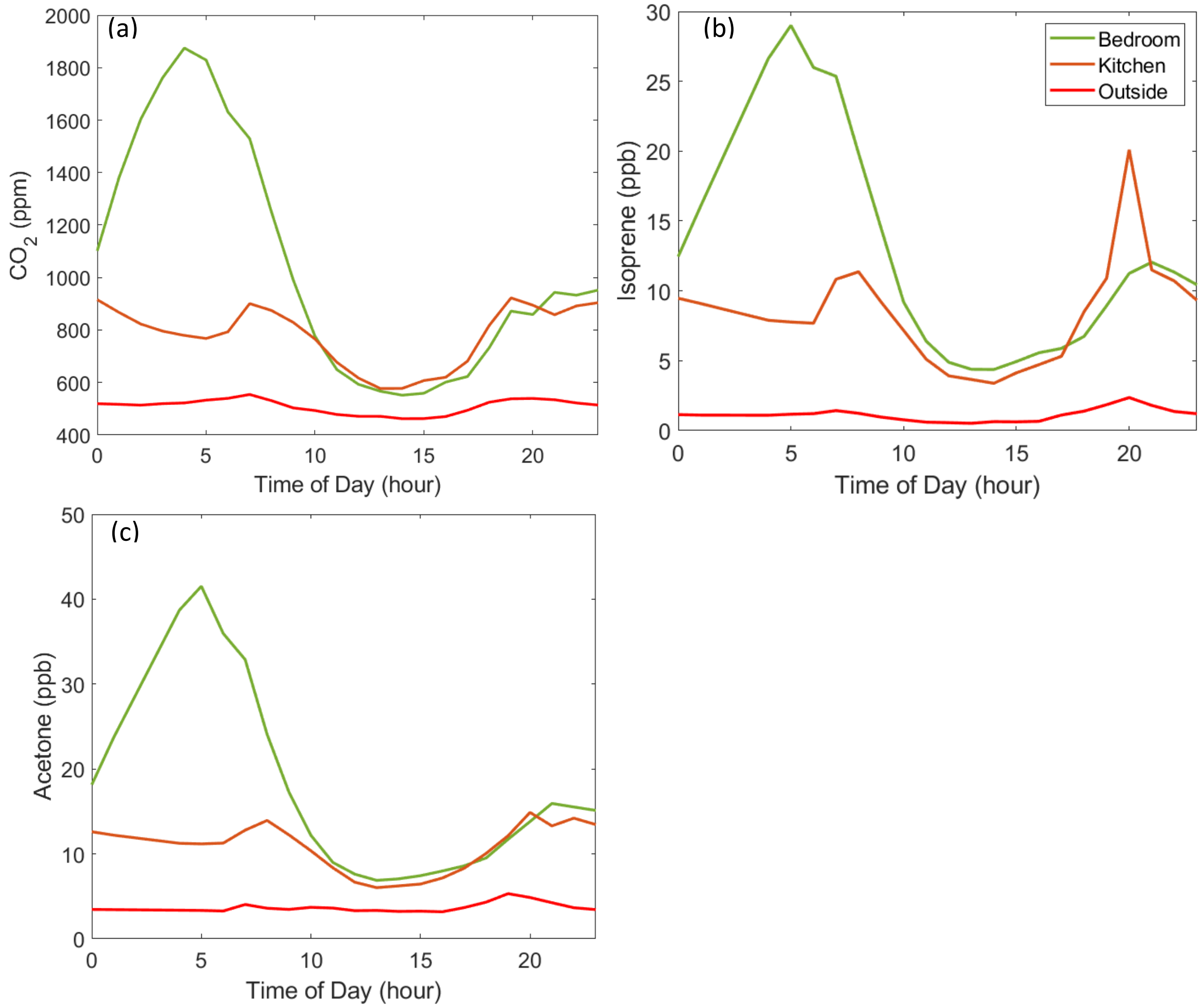

Figure S4. Diel plots of mean (a) CO_2, (b) isoprene, and (c) acetone concentrations in the bedroom (green), kitchen (orange), and outside (red) for the twelve nights and corresponding days as enumerated in Table S3.

Carbon dioxide is generally a reliable tracer of human breath emissions. To probe that assumption, a correlation analysis like that applied for CO_2 measurements was also performed with respect to both isoprene and acetone. They are the most abundant VOC bioeffluents detected and the best-correlated with CO_2. The comparison between correlations with respect to CO_2 (*x*-axis) and with respect to isoprene (*y*-axis) is displayed in Figure S5, which is color-coded in the same manner as in Figure 3. The analysis for isoprene revealed that 33 compounds had $R \geq 0.70$, but only those that overlap with the results from the analysis for CO_2 are considered in the figure. With the obvious exception of CO_2 itself and $C_7H_7^+$, every compound considered has an equal or higher R value with respect to isoprene, highlighted by the fact that they are all on or above the 1:1 line. This better correlation with isoprene suggests that, at least in this residence, isoprene provides a more reliable breath emission tracer than CO_2. One possible reason for this finding is that the isoprene signal does not encounter significant interference from outdoor signals at night, whereas

the CO_2 signal is elevated outdoors at night, likely due to a combination of respiration from soils and plants and accumulation of emissions into a more stable atmosphere (than during daytime) from burning carbonaceous fuels.

The higher number of compounds showing $R \geq 0.70$ with respect to isoprene than with respect to CO_2 offers further evidence of isoprene being more reliable as a VOC bioeffluent tracer. No compounds appeared to be strongly anti-correlated with isoprene. The correlation analysis for acetone revealed that fewer compounds have values of $R \geq 0.70$ than noted for CO_2 or isoprene, indicating that it is not as reliable a bioeffluent tracer. This outcome is expected given acetone's broader range of potential sources. No VOC signals had an R value greater than 0.70 or less than -0.70 with respect to temperature, but nine compounds showed strong correlation with relative humidity. Of the more abundant compounds, only acetone had a relevant R value (0.71) with respect to relative humidity. Hexanal and some alkyl fragments also had high R values, indicating a potential role of relative humidity in the fragmentation of some VOCs during PTR analysis.

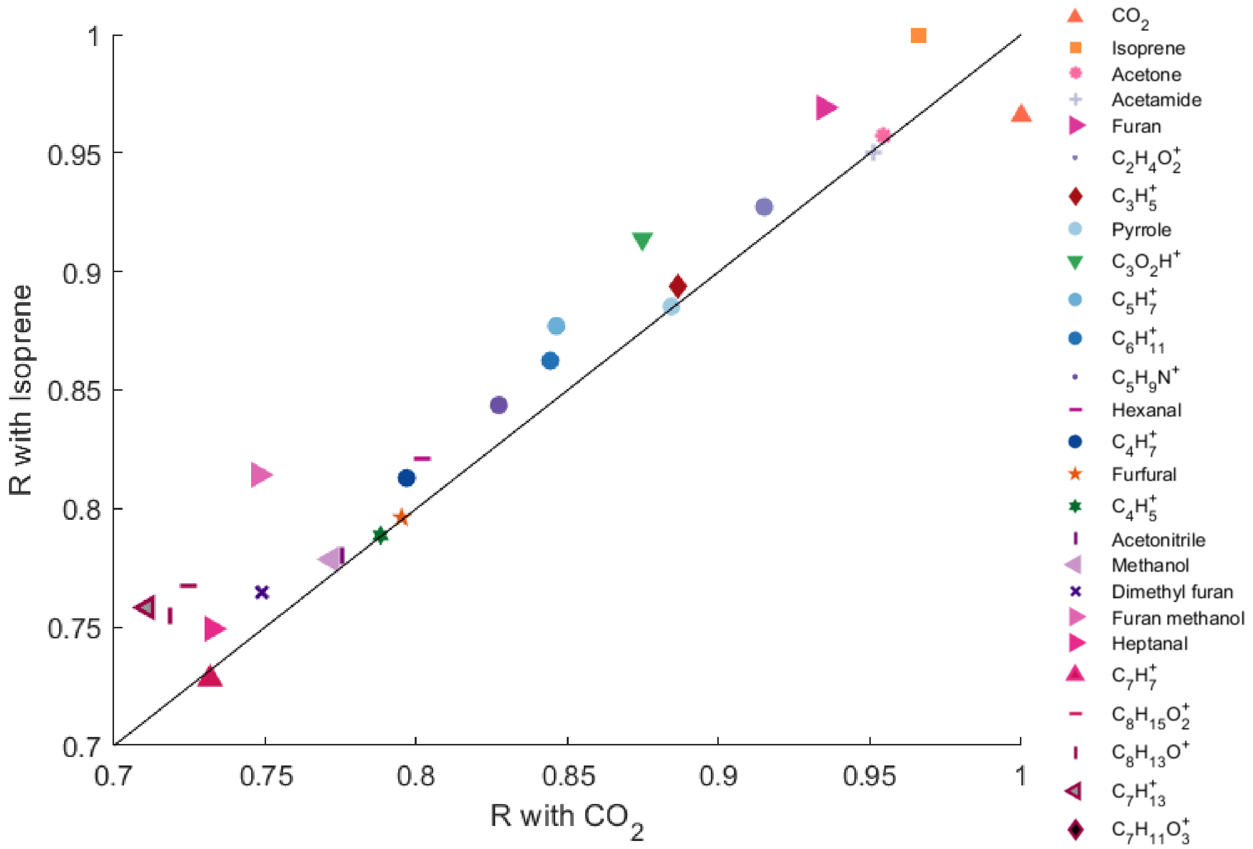

Figure S5. Scatter plot of compounds with values of $R \geq 0.70$ with respect to CO_2 (*x*-axis) and isoprene (*y*-axis). Some markers may overlap. The black line indicates a 1:1 relationship for the correlation coefficients.

Bedroom VOC Emission Rates

Table S5. Average upper (air exchange with outdoors) and lower (air exchange with kitchen) bounds for the emission rates (μg h^{-1}) of all 99 VOC species included in this analysis in the bedroom over 12 nights at H3 using full occupancy period.

m/z	Ion Formula	Name	ER_{upper} (mean ± SD)	ER_{lower} (mean ± SD)
41.038	$C_3H_5^+$	alkyl fragment	52 ± 24	20 ± 17
49.016	CH_5S^+	methanethiol	2.0 ± 1.1	-
51.007			1.2 ± 1.0	0.42 ± 0.51
53.038	$C_4H_5^+$	alkyl fragment	1.3 ± 0.5	0.75 ± 0.54
55.039			69 ± 20	49 ± 32
55.053	$C_4H_7^+$		64 ± 26	35 ± 9.1
59.049	$C_3H_7O^+$	acetone	1530 ± 440	1120 ± 500
60.020	$C_2H_4O_2^+$	a	4.3 ± 1.8	2.8 ± 1.7
60.051	$C_2H_6NO^+$	acetamide	48 ± 18	44 ± 20
64.030			0.90 ± 0.54	0.04 ± 1.40
65.024	$CH_5O_3^+$		6.0 ± 4.1	1.80 ± 0.96
65.034	$C_5H_5^+$		3.3 ± 1.8	1.0 ± 1.0
65.086			1.70 ± 1.50	0.46 ± 0.83
67.053	$C_5H_7^+$	alkyl fragment	7.1 ± 3.3	4.9 ± 1.9
68.051	$C_4H_6N^+$	pyrrole	3.5 ± 1.2	2.3 ± 1.1
68.995	$C_3O_2^+$		42.4 ± 0.9	1.8 ± 1.0
69.033	$C_4H_5O^+$	furan	25 ± 8.8	11.0 ± 10.0
69.069	$C_5H_9^+$	isoprene	1030 ± 330	890 ± 310
70.036			1.1 ± 0.6	0.64 ± 1.20
71.049	$C_4H_7O^+$	MVK + isomers	63 ± 27	16 ± 44
71.085	$C_5H_{11}^+$	pentene	18.6 ± 9.0	3.5 ± 8.9
74.032			1.70 ± 1.00	-
76.024			1.10 ± 0.87	0.34 ± 0.91
77.033	$C_3H_9S^+$	propanethiol	2.9 ± 1.8	1.40 ± 0.84
80.040			2.8 ± 1.2	1.30 ± 1.40
81.036	$C_5H_5O^+$		18.8 ± 9.6	2.8 ± 9.0
83.049	$C_5H_7O^+$	furan methanol	21 ± 9	1.7 ± 9.6
83.085	$C_6H_{11}^+$	alkyl fragment	110 ± 49	65 ± 29
84.051	$C_4H_5NO^+$		1.70 ± 0.97	0.54 ± 0.67
84.088	$C_5H_9N^+$		8.0 ± 3.4	4.8 ± 1.6
85.028	$C_4H_5O_2^+$	furanone	7.9 ± 5.0	1.4 ± 6.4
85.064	$C_5H_9O^+$	cyclopentanone + isomers	14.6 ± 8.3	6.4 ± 2.2
85.100	$C_6H_{13}^+$	hexene	6.5 ± 4.4	2.5 ± 1.9
86.061	$C_4H_8NO^+$		1.2 ± 1.3	1.3 ± 2.2
87.044	$C_4H_7O_2^+$	diacetyl?	24 ± 16	-
88.044	$C_4H_8S^+$		2.4 ± 1.9	0.39 ± 1.50
88.075	$C_4H_{10}NO^+$		1.40 ± 0.58	-
89.043	$C_3H_5O_3^+$		15.4 ± 6.2	6.6 ± 11.3
90.047			0.86 ± 0.67	0.38 ± 1.60
91.053	$C_7H_7^+$		12.7 ± 5.0	9.5 ± 5.4
93.039	$C_6H_5O^+$	aromatic frag?	9.1 ± 6.8	3.3 ± 1.7
97.063	$C_6H_9O^+$	dimethyl furan	22 ± 14	4.3 ± 5.4
97.100	$C_7H_{13}^+$		23 ± 13	8.9 ± 1.9
101.026	$C_4H_5O_3^+$	succinic anhydride	3.2 ± 2.0	-
101.060	$C_5H_9O_2^+$	4-OPA	18.7 ± 9.8	2.7 ± 6.4
101.096	$C_6H_{13}O^+$	hexanal	13.4 ± 6.6	4.0 ± 2.7

102.091			2.0 ± 0.9	1.30 ± 0.92
103.057	$C_4H_7O_3^+$	acetate anhydrate	8.7 ± 4.7	0.8 ± 8.2
104.056	$C_7H_6N^+$	benzonitrile	0.75 ± 0.51	0.30 ± 0.52
105.034	$C_4H_9OS^+$	methional	3.1 ± 1.6	1.6 ± 2.1
107.050	$C_7H_7O^+$	benzaldehyde	76 ± 44	56 ± 39
109.099	$C_8H_{13}^+$	6-MHO fragment	17.9 ± 10.4	10.4 ± 7.6
110.066			0.92 ± 0.73	0.56 ± 1.00
111.081	$C_7H_{11}O^+$		7.3 ± 4.6	1.8 ± 2.8
111.116	$C_8H_{15}^+$	1-octen-3-ol frag + others	14.8 ± 7.3	6.0 ± 2.7
113.046			0.83 ± 0.93	0.05 ± 0.58
113.096	$C_7H_{13}O^+$		3.7 ± 2.1	-
115.076	$C_6H_{11}O_2^+$		4.6 ± 2.7	0.9 ± 2.3
115.112	$C_7H_{15}O^+$	heptanal	8.1 ± 4.6	2.9 ± 1.4
117.089	$C_6H_{13}O_2^+$	hexanoic acid	7.7 ± 6.3	2.2 ± 2.4
119.049			2.9 ± 2.5	2.0 ± 3.0
119.083	$C_9H_{11}^+$		11.9 ± 15.5	9.3 ± 18.5
121.029	$C_7H_5O_2^+$		2.3 ± 1.7	1.40 ± 1.30
121.066	$C_8H_9O^+$	anisaldehyde	6.5 ± 3.9	3.2 ± 2.2
122.002			0.32 ± 0.84	0.1 ± 2.2
123.081			2.1 ± 1.5	0.62 ± 0.66
125.060	$C_7H_9O_2^+$		2.3 ± 0.8	1.4 ± 2.2
125.096	$C_8H_{13}O^+$		5.7 ± 3.2	3.2 ± 2.3
125.132	$C_9H_{17}^+$		8.8 ± 4.5	3.8 ± 1.3
127.001			2.3 ± 1.8	1.5 ± 1.0
127.077	$C_7H_{11}O_2^+$		2.7 ± 1.1	0.79 ± 1.40
127.112	$C_8H_{15}O^+$	6-MHO + others	58 ± 30	38 ± 25
129.128	$C_8H_{17}O^+$		5.2 ± 3.6	2.0 ± 2.2
131.995			0.18 ± 0.81	0.77 ± 1.60
136.022	$C_7H_6NS^+$	benzothiazole	3.2 ± 1.8	3.5 ± 2.1
137.060	$C_8H_9O_2^+$	4-anisaldehyde	6.6 ± 3.4	3.6 ± 4.5
137.132	$C_{10}H_{17}^+$	monoterpenes	470 ± 260	173 ± 178
139.078	$C_8H_{11}O_2^+$	creosol	2.0 ± 1.30	1.90 ± 1.50
139.111	$C_9H_{15}O^+$		5.3 ± 3.1	2.4 ± 0.7
139.149	$C_{10}H_{19}^+$		2.1 ± 1.4	1.10 ± 1.60
141.128	$C_9H_{17}O^+$	nonenal + others	1.70 ± 1.00	0.45 ± 1.40
143.088	$C_7H_{11}O_3^+$		2.9 ± 1.5	2.1 ± 1.5
143.110	$C_8H_{15}O_2^+$		5.3 ± 2.8	3.5 ± 2.3
143.145	$C_9H_{19}O^+$	C9 saturated carbonyl	910 ± 530	540 ± 175
153.015			1.90 ± 1.20	1.7 ± 2.7
153.055	$C_8H_9O_3^+$	vanillin + others	8.3 ± 7.4	10.0 ± 13.5
155.110	$C_9H_{15}O_2^+$		2.7 ± 1.9	2.2 ± 4.9
157.105	$C_8H_{13}O_3^+$		1.00 ± 1.20	1.00 ± 1.80
157.117	$C_9H_{17}O_2^+$		1.10 ± 0.83	0.79 ± 1.30
157.159	$C_{10}H_{21}O^+$	C10 saturated carbonyl	6.1 ± 3.3	4.3 ± 2.2
159.128	$C_8H_{19}O_2^+$		1.90 ± 1.30	0.50 ± 1.20
171.173	$C_{11}H_{23}O^+$	C11 saturated carbonyl	1.10 ± 1.40	0.52 ± 1.10
177.160	$C_{13}H_{21}^+$		0.53 ± 0.99	0.35 ± 1.10
371.102	$C_{10}H_{31}O_5Si_5^+$	D5 siloxane	147 ± 129	187 ± 250

Skin Emission Signals

Skin oil contains ~10% squalene which reacts with ozone to form specific products, including 6-MHO and 4-OPA [1]. Figure S6 shows the time series in the bedroom for ozone and these two squalene-ozonolysis products. There was no evidence to suggest that ozone concentrations were directly related to temperature or relative humidity in the bedroom. The average nighttime ozone concentration in the bedroom was low, 0.9 ± 0.5 ppb, but there was clear enhancement of ozone indoors during the day when ozone was also high outdoors. Aside from two notable peaks in the kitchen on Dec 01, 2021, as seen in panel (a) of Figure S6, the kitchen and bedroom concentration profiles are similar.

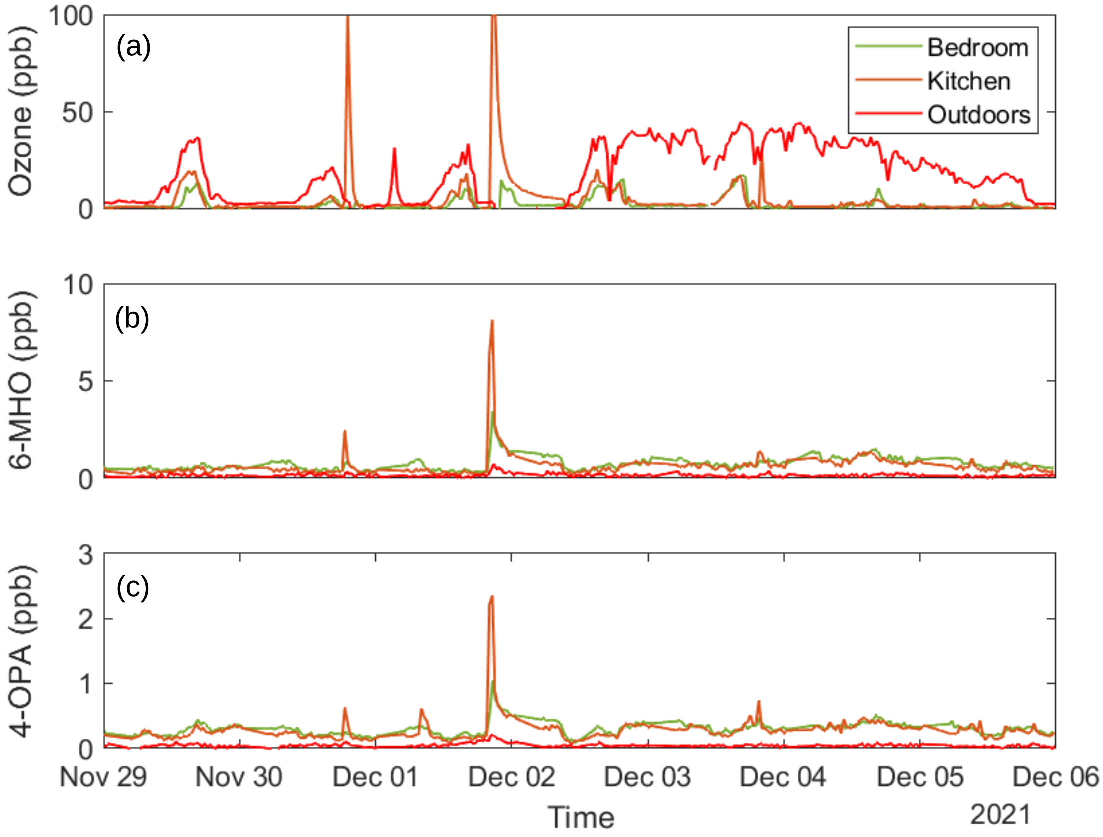

Figure S6. Time series of (a) ozone, (b) 6-MHO, and (c) 4-OPA in the bedroom (green), kitchen (orange), and outdoors (red) at H3 over one week. Midnight of each date included is indicated by tick marks on the *x*-axis.

Because skin oil oxidation products clearly have a different formation pathway than emissions from breath and can therefore be considered to have different sources within the human exposome, it is not appropriate to consider isoprene as a reliable tracer for this source category. Therefore, a correlation analysis with respect to ozone was undertaken to determine whether a skin emission tracer could be established. However, there was no evidence of correlation or anti-correlation with respect to ozone as the R values of VOC signals ranged from -0.35 to 0.41. Average 6-MHO and 4-OPA concentrations in the bedroom over the examined nighttime periods were 0.74 ± 0.20 ppb and 0.29 ± 0.07 ppb, respectively. As seen in panels (b) and (c) of Figure S6, indoor concentrations of both oxidation products are consistently enhanced indoors, with kitchen and bedroom

concentration profiles appearing similar. This finding is consistent with their production occurring mainly when ozone is elevated in the house during the day rather than during nighttime bedroom occupancy when the ozone level is very low. Peaks in ozone lead to faster production of 6-MHO and 4-OPA, as indicated by peaks occurring at the same time in all three profiles.

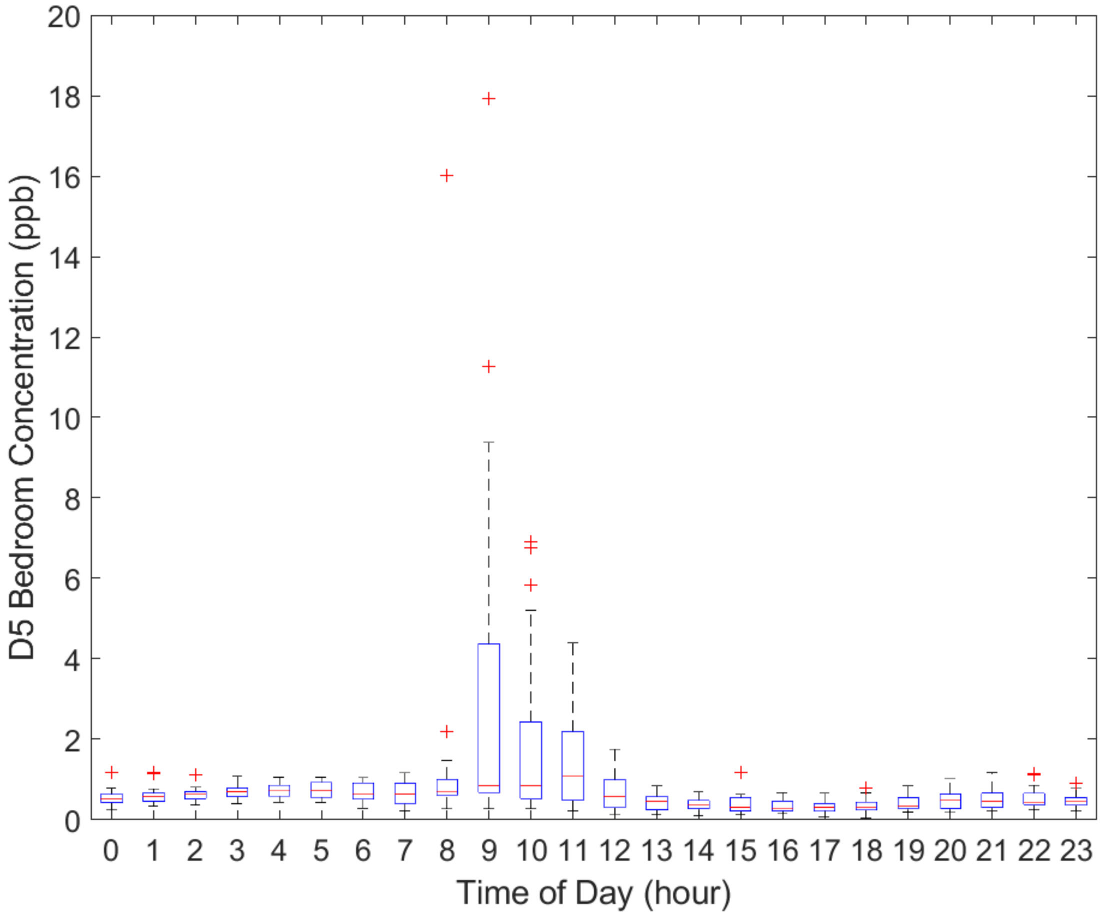

Figure S7. Diurnal profile, using the 12 nights and their corresponding days examined per Table S3, of D5 siloxane in the H3 bedroom. The top and bottom edges of the blue boxes represent the 75th and 25th percentiles, respectively, and the red lines inside the boxes represent the medians. The black whiskers extend distances of 1.5 times the interquartile range from the box edges. The red symbols denote outliers.

Cyclic volatile methyl siloxanes (cVMS, D3-D6) are emitted from some personal care products [2]. The D5 siloxane signal dominated the total observed cVMS contributions to indoor air composition, as expected from previous work. Average nighttime concentrations in the bedroom for D3-D6 siloxanes were 0.057 ± 0.018 ppb, 0.040 ± 0.015 ppb, 1.08 ± 0.63 ppb, and 0.101 ± 0.096 ppb, respectively. The peak levels of D3, D4, and D6 never exceeded 1 ppb in the bedroom during the time it was monitored, but D5 concentrations show peaks as high as ~ 20 ppb that occur most mornings. Figure S7 shows the D5 diurnal cycle in the bedroom, with a peak occurring at 09:00 and decaying throughout the morning. There is no evidence of emissions of D5 at night during sleeping periods. This pattern is likely the result of occupants regularly applying personal care products in the morning after waking up. There is no evidence of correlation between cVMS

and CO_2 or isoprene during the examined nighttime periods, which is expected as there is enough time for these compounds to volatilize off the skin throughout the day before occupants go to bed and there is no evidence from the time series or occupant activity logs suggesting a reapplication of products prior to sleeping. There is also no strong anti-correlation with ozone, indicating little to no oxidation, which is expected given that cVMS atmospheric lifetimes with respect to oxidation are much longer than the H3 bedroom air exchange rates [3, 4].

References

1. Liu, Y.; Misztal P. K.; Arata, C.; Weschler, C. J.; Nazaroff, W. W.; Goldstein, A. H. Observing ozone chemistry in an occupied residence. *Proceedings of the National Academy of Sciences of the U.S.A.* **2021**, *118*, e2018140118.
2. Molinier, B.; Arata, C.; Katz, E. F.; Lunderberg, D. M.; Liu, Y.; Misztal, P. K.; Nazaroff, W. W.; Goldstein, A. H. Volatile Methyl Siloxanes and Other Organosilicon Compounds in Residential Air. *Environ. Sci. Technol.* **2022**, *56*, 15427–15436.
3. Gkatzelis, G. I.; Coggon, M. M.; McDonald, B. C.; Peischl, J.; Aikin, K. C.; Gilman, J. B.; Trainer, M.; Warneke, C. Identifying Volatile Chemical Product Tracer Compounds in U.S. Cities. *Environ. Sci. Technol.* **2021**, *55*, 188–199.
4. Weschler, C. J. Ozone in Indoor Environments: Concentration and Chemistry. *Indoor Air* **2000**, *10*, 269–288.
